# Canonical ligand-dependent and non-canonical ligand-independent EphA2 signaling in the eye lens of wild-type, knockout, and aging mice

**DOI:** 10.18632/aging.206144

**Published:** 2024-10-25

**Authors:** Jenna L. Horner, Michael P. Vu, Jackson T. Clark, Isaiah J. Innis, Catherine Cheng

**Affiliations:** 1 School of Optometry and Vision Science Program, Indiana University, Bloomington, IN 47405, USA

**Keywords:** fiber cells, epithelial cells, Y588, Y589, S897, phosphorylation, maturation, ephrin

## Abstract

Disruption of Eph-ephrin bidirectional signaling leads to human congenital and age-related cataracts, but the mechanisms for these opacities in the eye lens remain unclear. Eph receptors bind to ephrin ligands on neighboring cells to induce canonical ligand-mediated signaling. The EphA2 receptor also signals non-canonically without ligand binding in cancerous cells, leading to epithelial-to-mesenchymal transition (EMT). We have previously shown that the receptor EphA2 and the ligand ephrin-A5 have diverse functions in maintaining lens transparency in mice. Loss of ephrin-A5 leads to anterior cataracts due to EMT. Surprisingly, both canonical and non-canonical EphA2 activation are present in normal wild-type lenses and in the ephrin-A5 knockout lenses. Canonical EphA2 signaling is localized exclusively to lens epithelial cells and does not change with age. Non-canonical EphA2 signaling is in both epithelial and fiber cells and increases significantly with age. We hypothesize that canonical ligand-dependent EphA2 signaling is required for the morphogenesis and organization of hexagonal equatorial epithelial cells while non-canonical ligand-independent EphA2 signaling is needed for complex membrane interdigitations that change during fiber cell differentiation and maturation. This is the first demonstration of non-canonical EphA2 activation in a non-cancerous tissue or cell and suggests a possible physiological function for ligand-independent EphA2 signaling.

## INTRODUCTION

Erythropoietin-producing hepatocellular carcinoma (Eph) receptors make up the largest family of human receptor tyrosine kinases (RTKs) [1–3]. Expressed in nearly every cell in the body, Eph receptors are known to play extensive roles in cellular development and patterning [4, 5], proliferation [6–8], differentiation [9–11], motility [12], migration [13, 14], adhesion and repulsion [5, 15], as well as many other physiological roles (summarized in [16]). Human Eph receptors are divided into two subclasses, EphAs (9 receptors) and EphBs (5 receptors), based on sequence similarity and ligand affinity [5, 17, 18]. A special class of membrane-bound ephrin ligands bind to Eph receptors, and these ligands are also divided into two subclasses, ephrin-As (5 ligands) that are glycosylphosphatidylinositol-anchored ligands, and ephrin-Bs (5 ligands) that are transmembrane ligands with a cytosolic tail [2, 5, 16, 17, 19–22]. Eph receptors and ephrin ligands bind promiscuously, and while most interactions are EphA to ephrin-A or EphB to ephrin-B, cross interactions between receptor and ligand subclasses can occur [5, 14, 16, 20]. Though these interactions are less common, EphA4 and EphB2 can bind ephrin-Bs and ephrin-As, respectively [20].

Recently, Eph-ephrin bidirectional signaling has been linked to roles in eye lens transparency (reviewed in [16]). The lens is an ellipsoid, self-contained, and transparent organ in the anterior chamber of the eye and is required for fine focusing of light to transmit a clear image onto the retina, the neural tissue in the back of the eye. Mutations in the *EPHA2* (encodes for the receptor EphA2) and *EFNA5* (encodes for the ligand ephrin-A5) genes have been linked to congenital and age-related cataracts in human patients [23–34]. Cataracts, defined as any opacity in the normally transparent lens, remain the leading cause of blindness in the world, and the mechanisms for cataracts caused by dysfunction of Eph-ephrin signaling remain unclear.

The lens is composed of two cell types, epithelial and fiber cells (Supplementary Figure 1). A monolayer of epithelial cells covers the anterior hemisphere of the lens, and the bulk of the lens is made up of specialized fiber cells that differentiated from epithelial cells [35]. Anterior epithelial cells remain quiescent and do not normally divide. Epithelial cells at the lens equator divide and differentiate into secondary fiber cells. Secondary fiber cells elongate toward the anterior and posterior poles of the lens and are added in concentric shells overlaid on previous generations of fiber cells [35]. Lens fiber cells are an elongated hexagonal shape in cross section with 2 broad sides and 4 short sides. To maintain optical transparency, the newly added peripheral fiber cells must undergo maturation, where the cells lose their nuclei and organelles [36]. Mature lens fibers without cellular organelles have very low metabolism, and the central, or nuclear, fiber cells contain high concentrations of crystallin proteins, which maintain the high refractive index of the lens center or nucleus [35]. The lens continues to grow throughout life by adding new layers of secondary fiber cells. A collagen capsule surrounding the lens prevents the shedding or loss of old fiber cells with age [35]. The lens presents a unique tissue where it is possible to study cells made in an aged tissue as well as chronologically old cells that have been present since the organism was an embryo at the center of the tissue.

Our work and the work of others show that EphA2 is primarily expressed in the equatorial epithelial cells and fiber cells, while ephrin-A5 is enriched in epithelial cells and at the anterior tips of lens fiber cells [16, 37–40]. *EphA2* knockout (KO or *^-/-^*) mice in the C57BL/6J background often develop mild nuclear opacities and exhibit abnormal equatorial lens epithelial cells organization that subsequently results in mispatterned fiber cells (Supplementary Figure 2) [38–42]. Loss of EphA2 leads to changes in inner and perinuclear fiber cell membrane morphology defects that are correlated with changes in lens nucleus size and stiffness [43]. *Ephrin-A5^-/-^* mice in the C57BL/6J background are known to develop an anterior polar cataract due to abnormal epithelial-to-mesenchymal transition (EMT) in normally quiescent anterior epithelial cells (Supplementary Figure 2) [16, 38, 39]. The anterior lens epithelial cells also displayed abnormal punctate E-cadherin staining, revealing potential defects within adherens junctions and cellular adhesions [39]. It is thought that these defects are what lead to the development of EMT, and the subsequent invasion of knockout epithelial cells into the underlying fiber cells [39].

EphA2 is one of two Eph receptors capable of signaling canonically and non-canonically [12]. Canonical or ligand-dependent Eph receptor signaling influences many physiological processes throughout development and aging, such as actin cytoskeleton regulation [41, 44, 45], cellular adhesions [46, 47], angiogenesis [48], and tissue patterning [49–51] and is generally associated with tumor suppression [1, 52–55]. In canonical or ligand-dependent signaling, binding of the ephrin ligand from one cell to the Eph receptor on a neighboring cell causes receptor-receptor dimerization, autophosphorylation of tyrosine residues, and bidirectional activation of both the ligand-bearing and the neighboring receptor-bearing cells [16, 56, 57]. In human EphA2, two conserved tyrosine residues Y588 (Y589 in mouse) and Y594 (Y595 in mouse) [58] have been identified as major phosphorylation sites for canonical EphA2 signaling [53, 59]. Since both the receptor and the ligand are membrane-bound, cell-cell interactions are required for facilitation of canonical signaling. Eph receptors can further oligomerize into large signaling centers located within lipid rafts in the plasma membrane [5].

Non-canonical signaling of EphA2 is ligand-independent activation of the receptor. Only EphA2 and EphA3 are known to signal non-canonically [12, 60, 61]. EphA2 is normally expressed in rapidly proliferating cells and has previously been studied extensively for its roles in tumor metastasis and carcinogenesis [4, 12, 16, 52, 53, 56, 62–67]. While EphA2 is overexpressed in many types of cancer, canonical phosphorylation of EphA2 is often reduced due to insufficient cell-cell contact or due to decreased expression of the ephrin-A1 ligand [4, 52, 63, 68]. In instances where a ligand cannot be bound, EphA2 receptors will dimerize and mediate downstream signaling changes through the phosphorylation of the EphA2 serine 897 (S897) (S898 in mouse) residue by basophilic AGC protein kinases (Akt, PKA, and RSK) [52, 56, 69, 70]. Non-canonical EphA2 signaling has been associated with driving melanoma cell phenotypes [71], enhanced levels of aggression in gliomas [4], tumor progression in several cancers [4, 12, 52, 62, 64, 72], metastasis [4, 53, 62], and EMT [4, 53, 70].

We investigated whether EphA2 canonical or non-canonical signaling are active in the lens and whether non-canonical EphA2 signaling is involved in the EMT phenotype of *ephrin-A5^-/-^* lens epithelial cells. Surprisingly, Western blots and immunostaining revealed the presence of both canonical and non-canonical EphA2 activation in control *ephrin-A5^+/+^*, and *EphA2^+/+^* lenses with a mild increase in *ephrin-A5^-/-^* lenses. Canonical EphA2 signaling is restricted to lens epithelial cells with increased staining in equatorial epithelial cells, and the level of ligand-dependent EphA2 activation does not change in level with age or in *ephrin-A5^-/-^* lenses. In contrast, non-canonical EphA2 activation dramatically increased with age despite decreased total EphA2 protein levels with age. EphA2 ligand-independent activation is also mildly increased in *ephrin-A5^-/-^* lenses. Immunostaining studies localize non-canonical EphA2 activation to equatorial epithelial cells and mature lens fiber cells. The localization does not appear to be obviously different with age or genotype. Our work suggests that canonical EphA2 signaling is important for maintaining lens epithelial cells while non-canonical EphA2 signaling may play a role in fiber cell maturation. This is the first report of non-canonical EphA2 activation in normal tissue and suggests that non-canonical EphA2 signaling can play a physiological, rather than a pathological, role.

## RESULTS

### Total EphA2 protein level decreases with age while non-canonical EphA2 activation increases with age and in *ephrin-A5^-/-^* lenses

To determine what EphA2 signaling pathways are activated in the lens, we performed capillary electrophoresis Western blots on protein lysates from lens epithelial cells and cortical fiber cells isolated of 6-week-old, 4-month-old, and 8-month-old *EphA2^+/+^*, *EphA2^-/-^*, *ephrin-A5^+/+^*, and *ephrin-A5^-/-^* mice. We chose these ages of mice to represent young, adult, and middle-aged animals. Mice develop age-related cataracts at approximately 10 months of age [73], and thus, we did not collect older samples for this study because it would be difficult to separate the effects of very old age from the changes due to the KO. *EphA2^-/-^* samples were used to verify antibody specificity (Supplementary Figure 3). We did not investigate the nuclear fiber cell fractions because we previously demonstrated that nuclear fiber cells do not have detectable levels of EphA2 [43]. The trends seen in *EphA2^+/+^* samples are comparable with *ephrin-A5^+/+^* samples.

**Figure 1:**
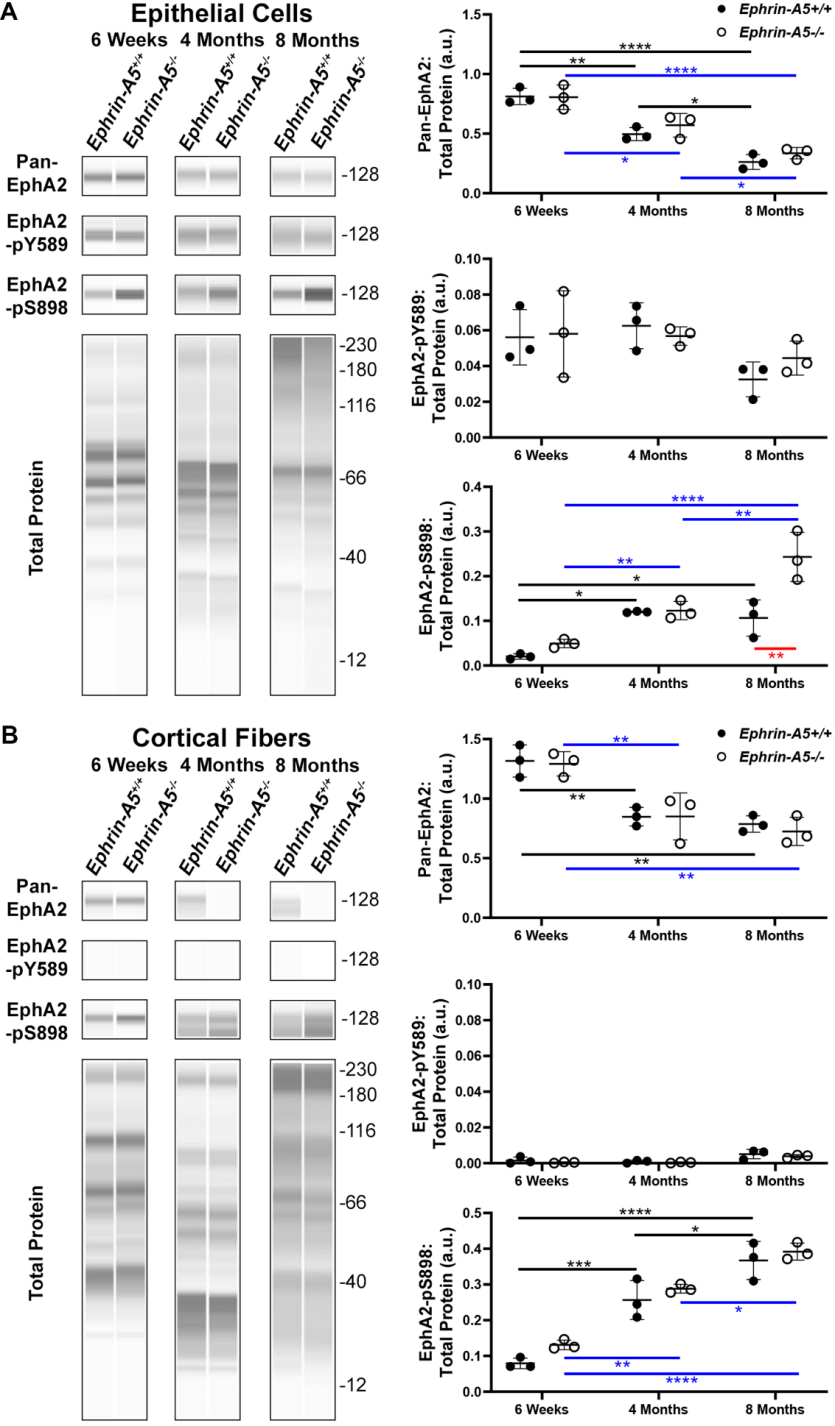
**JESS capillary-based Westerns demonstrate canonical EphA2-pY589 activation is restricted to the lens epithelial cells while non-canonical EphA2-pS898 phosphorylation is present in epithelial cells and cortical fibers of 6-week-old, 4-month-old, and 8-month-old *ephrin-A5^+/+^* and *ephrin-A5^-/-^* mice.** Representative gel bands for pan-EphA2 (~128kDa), EphA2-pY589 (~128kDa), EphA2-pS898 (~128kDa), and total protein profiles (12-230kDa) in lens epithelial (**A**) and cortical fiber cell fractions (**B**) are shown. The dot plots display the average and standard deviation of amounts for proteins of interest normalized to the amount of total protein in each sample. Black horizontal lines mark changes between *ephrin-A5^+/+^* samples at different ages, and blue horizontal lines mark changes between *ephrin-A5^-/-^* samples at different ages. Red horizontal line marks changes between *ephrin-A5^+/+^* and *ephrin-A5^-/-^* samples. *, *P*< 0.05; ** *P* < 0.01; *** *P* < 0.001; **** *P* < 0.0001. N = 3 biological replicates per group. (**A**) In epithelial cells, the amount of pan-EphA2 protein decreases with age, and the levels are comparable between *ephrin-A5^+/+^* and *ephrin-A5^-/-^* samples. Canonically active EphA2-pY589 protein is detected in all samples, and there is no change with age or genotype. Unexpectedly, non-canonically active EphA2-pS898 protein is detected in both *ephrin-A5^+/+^* and *ephrin-A5^-/-^* samples. In *ephrin-A5^+/+^* epithelial cells, there is increase in EphA2-pS898 levels between 6-week-old and 4-month-old or 8-month-old samples, and there is no change between 4-month-old and 8-month-old control samples. In *ephrin-A5^-/-^* epithelial cells, there is continuous increase of EphA2-pS898 levels with age, and there is a significant increase in EphA2-pS898 level in 8-month-old *ephrin-A5^-/-^* samples compared to that in control samples. (**B**) In cortical fiber cells, pan-EphA2 levels decrease between 6-week-old and 4-month-old or 8-month-old samples, and there is no change between 4-month-old and 8-month-old control samples. There are no differences in pan-EphA2 levels between *ephrin-A5^+/+^* and *ephrin-A5^-/-^* samples. Canonically active EphA2-pY589 protein was not detected in cortical fiber cells. Non-canonically active EphA2-pS898 protein levels increase with age in *ephrin-A5^+/+^* and *ephrin-A5^-/-^* samples. The levels of EphA2-pS898 proteins are not changed by the loss of ephrin-A5. The amount of pan-EphA2 decreased after 6-weeks but EphA2-pS898 amounts increased with age.

In the ephrin-A5 control and KO samples, we measured the levels of pan-EphA2 (total), canonically active EphA2 phosphorylated at tyrosine 589 (pY589), and non-canonically active EphA2 phosphorylated at serine 898 (pS898) (Figure 1). Within the epithelial cell fraction (Figure 1A), Western blots showed significant decreases the level of pan-EphA2 with age, consistent with a previous report [24]. The loss of ephrin-A5 did not alter the levels of pan-EphA2 when compared to littermate controls. Canonically active EphA2-pY589 protein levels within the epithelium remained unchanged across ages and genotypes. Surprisingly, non-canonically active EphA2-pS898 protein was detected in normal control *ephrin-A5^+/+^* epithelial cells, and the level of EphA2-pS898 increased significantly between 6-week-old and 4-month-old and between 6-week-old and 8-month-old control samples. There was no further increase in EphA2-pS898 levels between 4 months and 8 months in control lens epithelial cells. Similarly, there was an increase in EphA2-pS898 levels in *ephrin-A5^-/-^* lens epithelial cells with age, and unlike the control, in 8-month-old *ephrin-A5^-/-^* lens epithelial cells, we detected much higher levels of non-canonical activation. In samples from 4-month-old and 8-month-old ephrin-A5 control and KO mice, we noticed an increase in the height of the bands for pan-EphA2, EphA2-pY589, and EphA2-pS898 on the Western results. Presumably, this is due to cleavage and post-translational modifications of EphA2 [74].

Within the cortical fiber cells (Figure 1B), the pan-EphA2 protein decreased between 6-week-old and 4-month-old and between 6-week-old and 8-month-old control and KO samples. However, there was no further decrease in pan-EphA2 levels between 4 and 8 months of age. There was no difference in pan-EphA2 levels between ephrin-A5 control and KO age-matched samples. Canonically active EphA2-pY589 protein was absent from fiber cells of all ages. Non-canonically active EphA2-pS898 protein was found to be abundantly present in cortical fiber cells, and there was an obvious increase in EphA2-pS898 levels with age. Ephrin-A5 control and KO age-matched samples had comparable EphA2-pS898 levels. Similar to the epithelial cell sample Westerns, we observe an increase in the height of the bands for pan-EphA2 and EphA2-pS898 in the cortical fiber cell samples from 4-month-old and 8-month-old control and KO samples, presumably due to cleavage or post-translational modifications of EphA2 with age. These data show that canonical activation of EphA2 was restricted to the epithelial cells and does not change with age or genotype. Surprisingly, non-canonical activation of EphA2 is detected in ephrin-A5 control and KO epithelial cells and fiber cells. The level of pan-EphA2 decreased significantly with age, but there was an obvious increase in non-canonical EphA2 activation with age and in 8-month-old *ephrin-A5^-/-^* epithelial cells.

### Pan-EphA2 localizes along cell membranes throughout the lens

To further investigate the localization of these signaling pathways, immunostaining was performed on frozen lens sections in the longitudinal and cross orientations from 6-week-old, 4-month-old, and 8-month-old *ephrin-A5^+/+^* and *ephrin-A5^-/-^* mice. Sections were stained with pan-EphA2 antibody, EphA2-pY589 antibody, or EphA2-pS898 antibody and counterstained with phalloidin for F-actin and DAPI for cell nuclei. Antibody specificity was confirmed with *EphA2^-/-^* frozen sections (Supplementary Figure 4), and the staining signal between *EphA2^+/+^* and *ephrin-A5^+/+^* sections were consistent. The staining signals were comparable between sections from 6-week-old, 4-month-old, and 8-month-old *ephrin-A5^+/+^* and *ephrin-A5^-/-^* mice. For some data sets, we will only include the 4-month-old data in the main text as representative data, and the 6-week-old and 8-month-old data can be found in the supplement.

**Figure 2:**
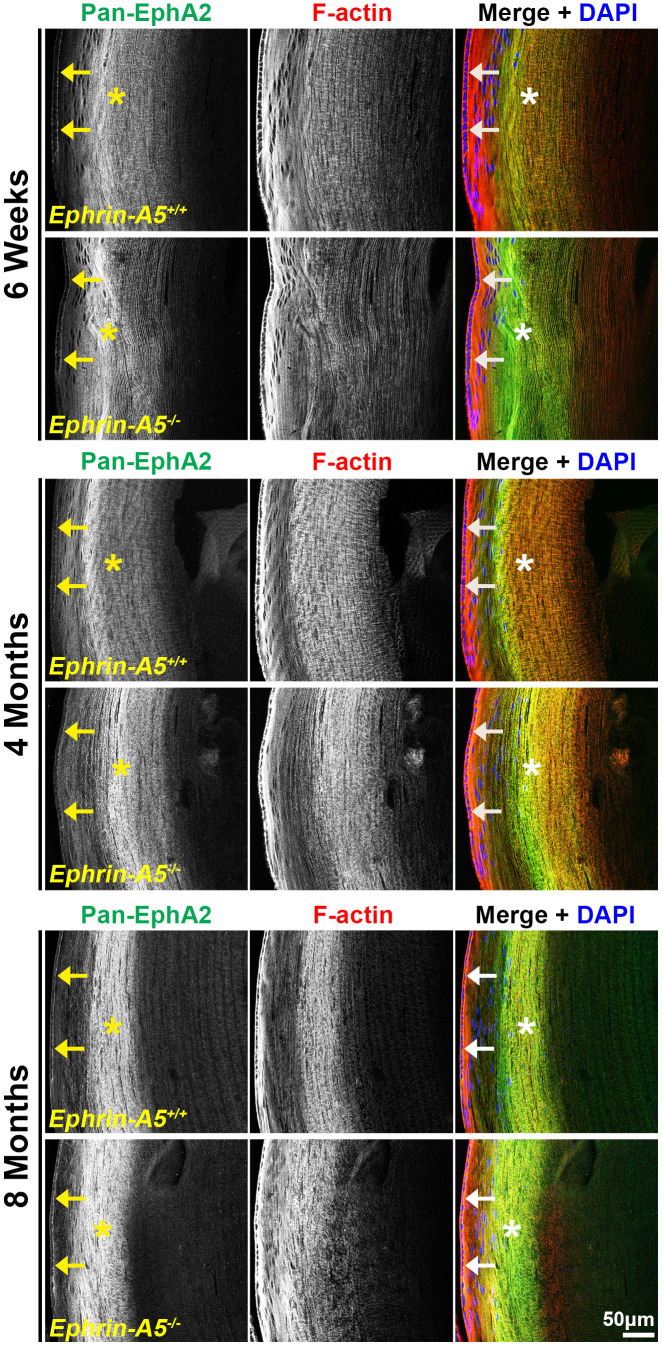
**Pan-EphA2 is localized to epithelial and fiber cells with enriched signal in fiber cells.** Longitudinal lens sections from 6-week-old, 4-month-old, and 8-month-old *ephrin-A5^+/+^* and *ephrin-A5^-/-^* mice were stained with pan-EphA2 (green) antibody, phalloidin (F-actin, red), and DAPI (nuclei, blue). The pan-EphA2 signal was visible in epithelial cells (arrows) and was strongly localized within the cortical fiber cell region (asterisks). This pattern remained consistent between age-matched controls and *ephrin-A5^-/-^* lenses. Scale bar, 50 μm.

**Figure 3:**
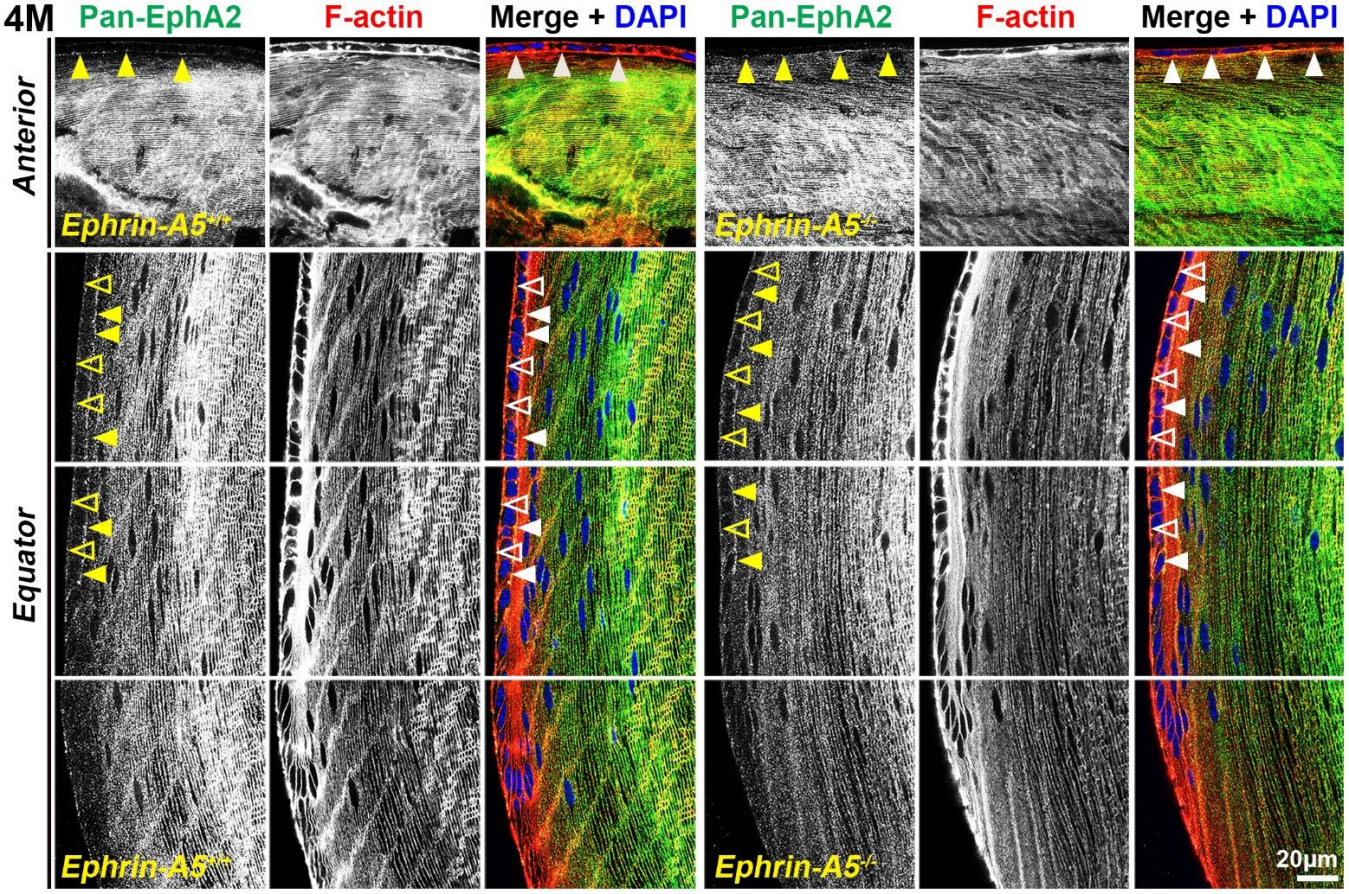
**In lens cells, pan-EphA2 is localized to the cell membrane.** Longitudinal lens sections from 4-month-old control and *ephrin-A5^-/-^* mice were stained with pan-EphA2 (green) antibody, phalloidin (F-actin, red), and DAPI (nuclei, blue). Images of the equator region were taken in sequence along similar areas of the lens. Fluorescence signal from pan-EphA2 appeared along the cell membrane within the lens. In anterior epithelial cells, there was increased pan-EphA2 signal at the apical-apical junction (arrowheads) between epithelial cells and fiber cells. In equatorial epithelial cells, pan-EphA2 was enriched at the basal (open arrowheads) membrane between epithelial cells and the lens capsule and at the apical membrane (arrowheads). In cortical lens fiber cells, pan-EphA2 outlined the cell membrane. There are no obvious differences between control and *ephrin-A5^-/-^* sections. Scale bars, 20 μm.

**Figure 4:**
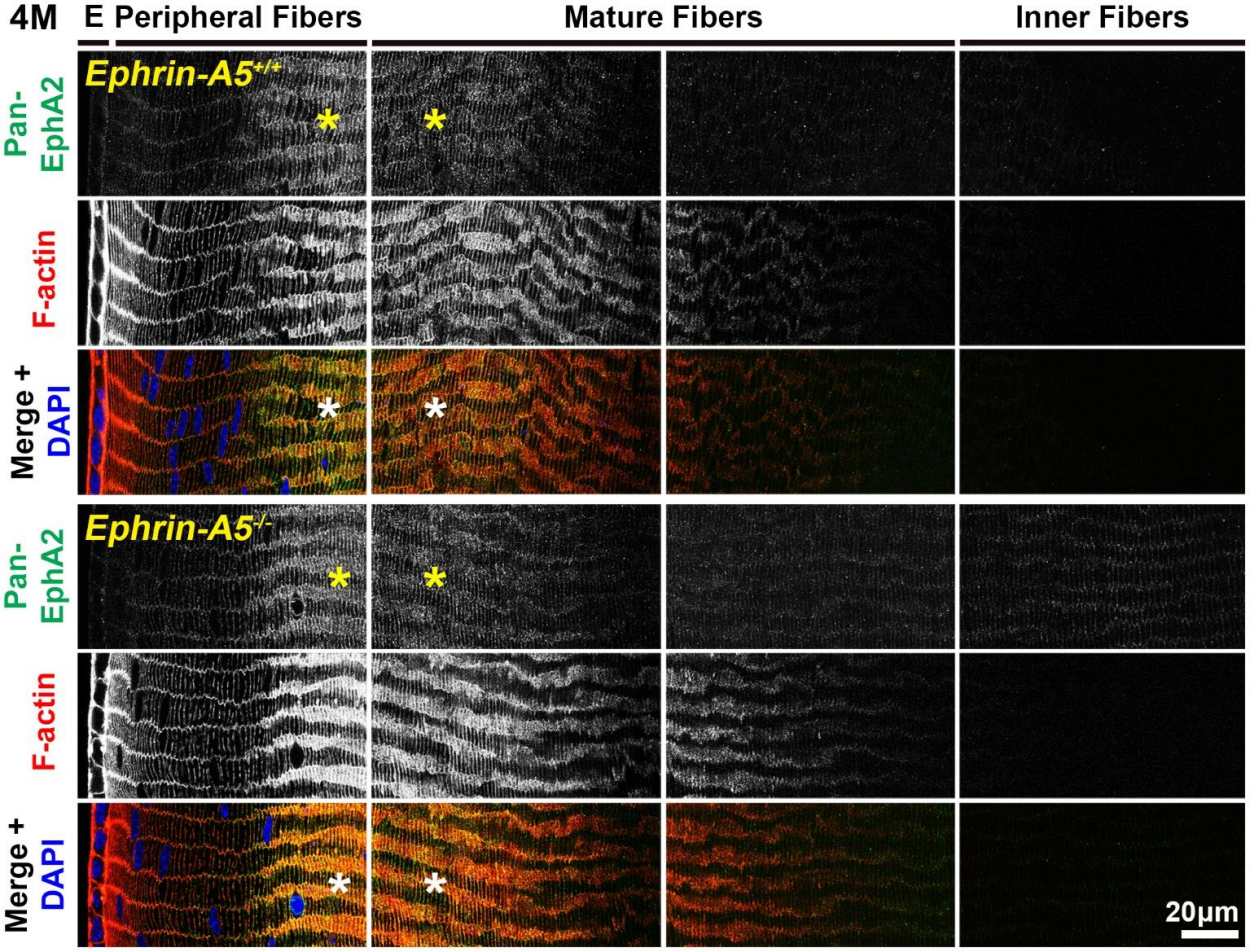
**In lens fiber cells, pan-EphA2 is localized to the cell membrane and enriched along the short sides.** Lens sections in the cross orientation from 4-month-old control and *ephrin-A5^-/-^* mice were stained with pan-EphA2 (green) antibody, phalloidin (F-actin, red), and DAPI (nuclei, blue). E denotes the epithelial cells, and images of the fiber cells were taken in sequence along similar areas of the lens equator. Fluorescence from pan-EphA2 was along the cell membrane of peripheral and mature fiber cells in the lens cortex region (asterisks). The signal was enriched along the short sides of lens fiber cells. Scale bar, 20 μm.

Immunostaining of longitudinal sections revealed that pan-EphA2 localized to epithelial cells (Figure 2, arrows) and fiber cells with a band of increased fluorescence between mature and inner fiber cells that remained consistent with age (Figure 2, asterisks). There were no obvious differences in the staining pattern for pan-EphA2 between control and ephrin-A5 KO sections. To better visualize the staining pattern in epithelial cells, images were taken with high magnification of longitudinal lens sections from 4-month-old *ephrin-A5^+/+^* and *ephrin-A5^-/-^* mice, which showed localization of the staining to be primarily along the apical-apical junction of anterior epithelial and fiber cells (Figure. 3, arrowheads) in control and KO sections. At the lens equator, the localization of EphA2 expands to both the apical and basal side of epithelial cells (Figure 3, arrowheads and open arrowheads, respectively). The location of the cell membrane was judged by the F-actin staining signal. There was enriched EphA2 staining signal along fiber cell membranes. Using cross orientation sections to examine fiber cell staining patterns in detail, we confirmed the localization of pan-EphA2 protein along the short and long sides of peripheral and mature fiber cell membranes, with slightly increased fluorescence detected on the short sides of the mature fiber cells (Figure 4, asterisks). The staining signal for pan-EphA2 is low in the inner fiber cells. These localization patterns in sections from 4-month-old mice were consistent in high magnification images of sections from 6-week-old (Supplementary Figures 5, 7) and 8-month-old control and KO mice (Supplementary Figures 6, 8).

### Canonically active EphA2-pY589 staining localizes to epithelial cells and is enriched at the lens equator

**Figure 5:**
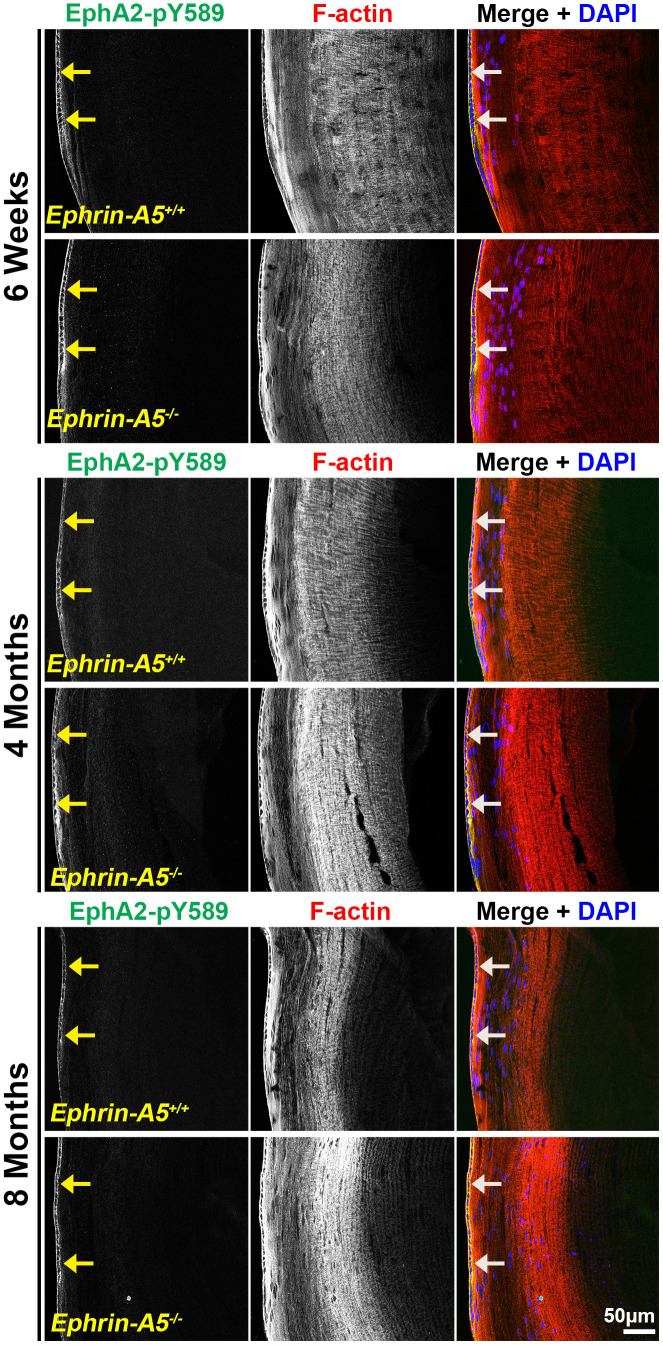
**Canonically activated EphA2-pY589 protein is localized exclusively in epithelial cells.** Longitudinal lens sections from 6-week-old, 4-month-old, and 8-month-old *ephrin-A5^+/+^* and *ephrin-A5^-/-^* mice were stained with EphA2-pY589 (green) antibody, phalloidin (F-actin, red), and DAPI (nuclei, blue). The EphA2-pY589 fluorescence highlighted the lens epithelial cells (arrows) across all age-matched control and *ephrin-A5^-/-^* lenses. Scale bar, 50 μm.

**Figure 6:**
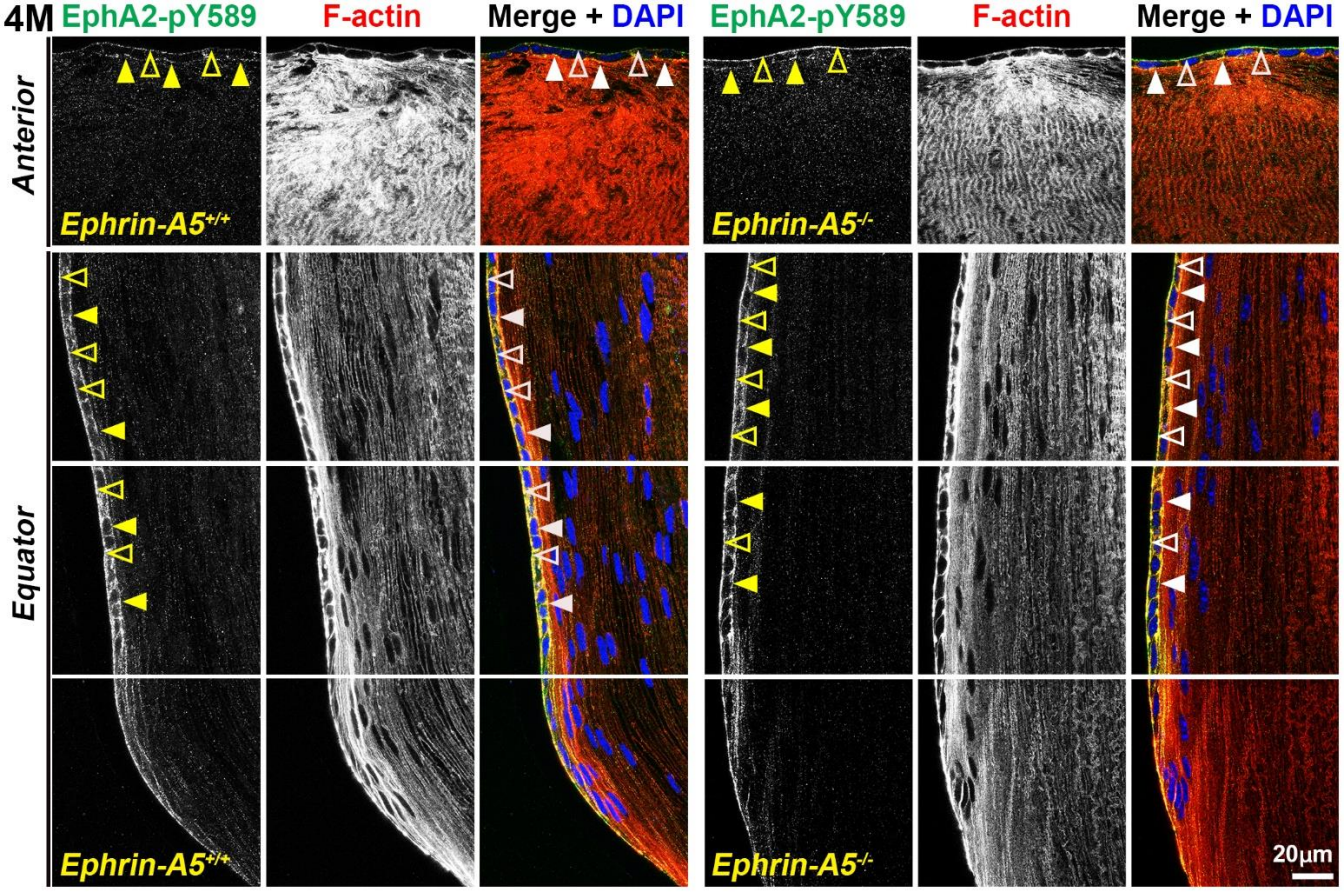
**Canonically active EphA2-pY589 protein is enriched at basal and apical membranes of lens epithelial cells.** Images of the equator region were taken in sequence along similar areas of the lens. In 4-month-old control and *ephrin-A5^-/-^* longitudinal lens sections stained with EphA2-pY589 (green) antibody, phalloidin (F-actin, red), and DAPI (nuclei, blue), the EphA2-pY589 signal was visible in anterior lens epithelial cells with enrichment at the basal (open arrowheads) and apical (arrowheads) membranes. There was increased EphA2-pY589 staining signal in equatorial epithelial cells with enrichment at the basal (open arrowheads) and apical (arrowheads) membranes. EphA2-pY589 staining was also visible between equatorial epithelial cells along the lateral membranes. There are no obvious differences in staining signal and localization between control and *ephrin-A5^-/-^* sections. Scale bar, 20 μm.

Next, we investigated where canonically active EphA2-pY589 signals were localized in the lens. Sections in the longitudinal orientation stained with EphA2-pY589 revealed fluorescence signals only within epithelial cells of control and KO lens sections (Figure 5, arrows). The staining pattern was very similar between control and KO sections in all age groups. Almost no signal was detected in the fiber cells, which is consistent with the Western blot results. When we examined epithelial cells in sections from 4-month-old *ephrin-A5^+/+^* and *ephrin-A5^-/-^* mice, there was much lower staining signal in the anterior epithelial cells compared to equatorial epithelial cells (Figure 6). The signal in anterior epithelial cells was mostly at the basal surface (Figure 6, open arrowheads) with weak staining at the apical-apical junction (Figure 6, arrowheads). At the lens equator, the stronger EphA2-pY589 staining signals were in equatorial epithelial cells along the apical and basal cell surfaces (Figure 6, arrowheads and open arrowheads, respectively) and between the cells at the lateral membranes. There appears to be brighter staining signal in the equatorial epithelial cells compared to that in the anterior epithelial cells. Because canonical EphA2 signaling was only detected in epithelial cells, staining of cross orientation sections, usually used to examine fiber cell membranes, is not shown. We observed similar epithelial cell staining patterns in 6-week-old (Supplementary Figure 9) and 8-month-old *ephrin-A5^+/+^* and *ephrin-A5^-/-^* lens sections (Supplementary Figure 10). While previous work has suggested the canonical ligand-mediated EphA2 signaling is likely to be active in the lens, this is the first direct evidence that bidirectional EphA2 signaling is active in lens epithelial cells.

### Non-canonically active EphA2-pS898 staining is enriched in mature fiber cells

**Figure 7:**
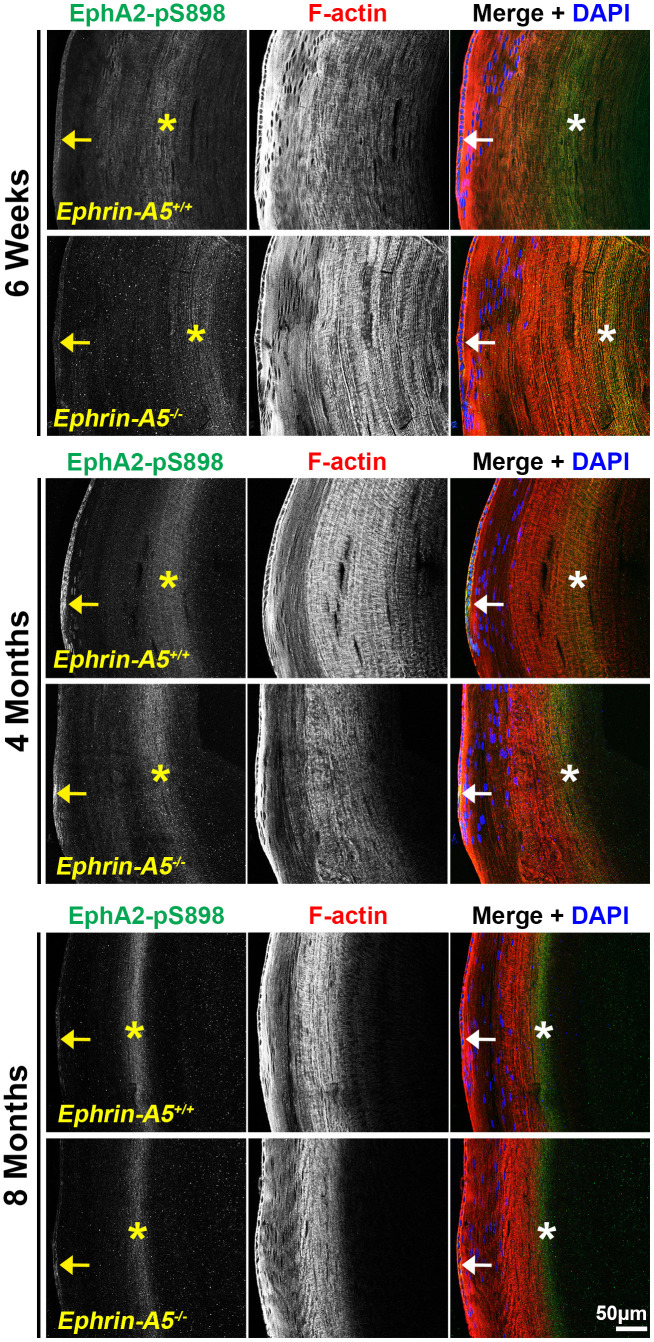
**Non-canonically activated EphA2-pS898 protein is localized in epithelial cells and mature fiber cells.** Longitudinal lens sections from 6-week-old, 4-month-old, and 8-month-old *ephrin-A5^+/+^* and *ephrin-A5^-/-^* mice were stained with EphA2-pS898 (green) antibody, phalloidin (F-actin, red), and DAPI (nuclei, blue). Fluorescence of EphA2-pS898 was found within the epithelial cells (arrows) and as a band within mature fiber cells in the lens cortex (asterisks). The staining pattern remained consistent between age-matched control and *ephrin-A5^-/-^* lenses. There was a noticeable narrowing and intensification of the EphA2-pS898 band of signal in the cortical fiber cells region with age, which is likely due to compression of fiber cells as the lens continues to grow. Scale bar, 50 μm.

Finally, we immunostained lens sections to reveal where non-canonical EphA2-pS898 signaling is active. In low magnification images of longitudinal sections from *ephrin-A5^+/+^* and *ephrin-A5^-/-^* mice, EphA2-pS898 staining showed localization to the epithelium (Figure 7, arrows) and a specific subset of mature fiber cells in the lens cortex (Figure 7, asterisks). The band of fluorescence in the mature fiber cells appeared more diffuse in 6-week-old sections, and the staining signal in those fibers compresses with age into a narrower band. Though the fluorescence signal in the fiber cells appeared to change with age, the same subset of mature cells was stained in sections from all ages. The EphA2-pS898-positive mature fiber cells are in a region where these cells have just eliminated their cellular organelles. With continued differentiation of the mature fibers as they are compacted toward the center of the lens, the EphA2-pS898 staining signal is lost. The compression of the fluorescence signal with age is due to the continuous addition of fiber cells in concentric shells in the lens cortex and the lens capsule that restricts the expansion of the tissue. This compression of signal can also be observed in the DAPI channel (blue) where the nuclei in the lens periphery were similarly compressed. There were no obvious differences between *ephrin-A5^+/+^* and *ephrin-A5^-/-^* lens sections in each age group.

**Figure 8:**
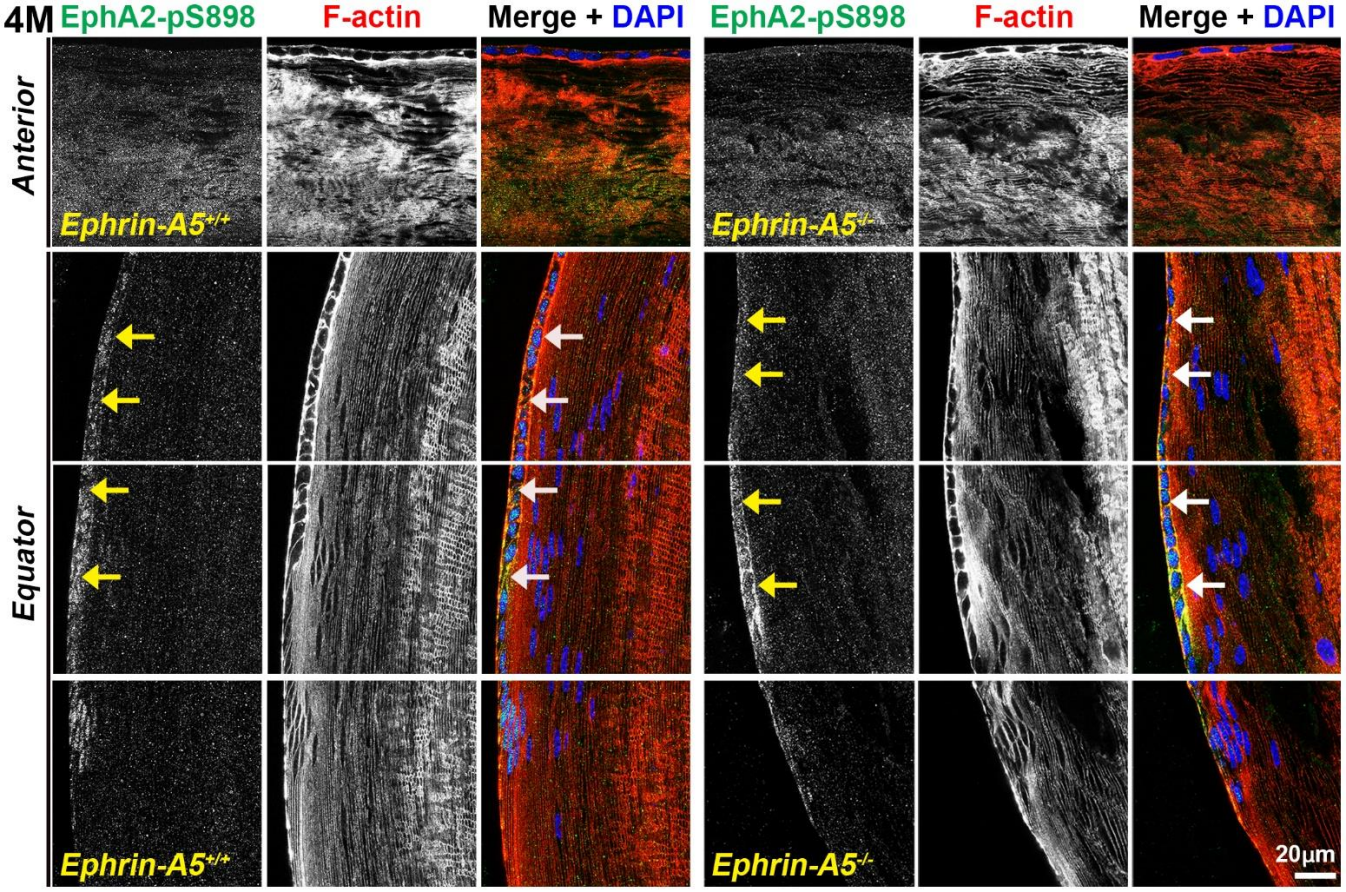
**Non-canonically active EphA2-pS898 protein is enriched in equatorial lens epithelial cells.** Longitudinal lens sections from 4-month-old control and *ephrin-A5^-/-^* mice were stained with EphA2-pS898 (green) antibody, phalloidin (F-actin, red), and DAPI (nuclei, blue). Images of the equator region were taken in sequence along similar areas of the lens. Mature fiber cells with strong EphA2-pS898 cannot be seen in these images of the peripheral fibers. EphA2-pS898 signal was found to weakly stain anterior epithelial cells, and there was increased signal in equatorial epithelial cells (arrows). There are no obvious differences between control and *ephrin-A5^-/-^* lenses. Scale bar, 20 μm.

**Figure 9:**
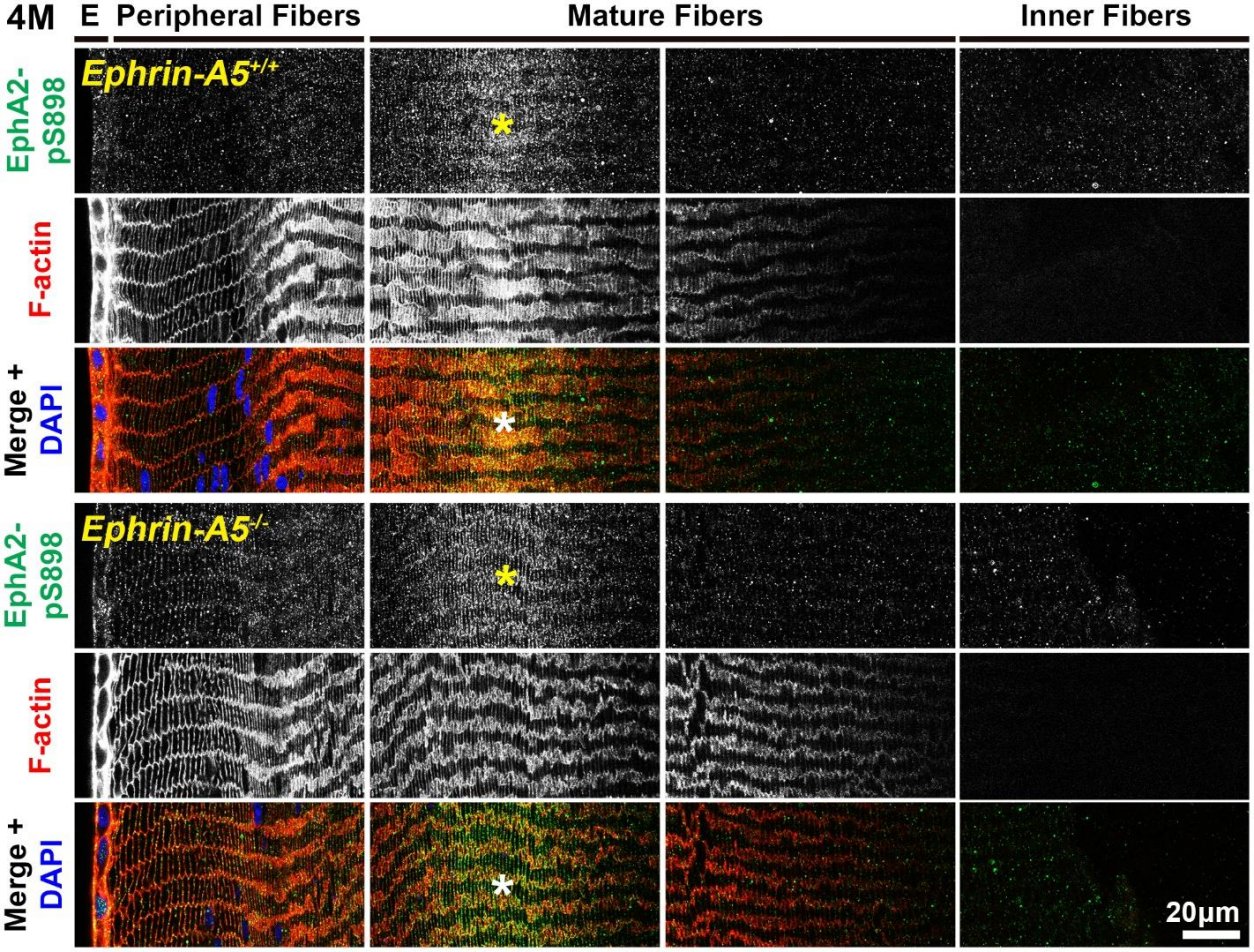
**In lens fiber cells, EphA2-pS898 signal is localized to the cell membrane of mature fibers.** Lens sections in the cross orientation from 4-month-old control and *ephrin-A5^-/-^* mice were stained with EphA2-pS898 (green) antibody, phalloidin (F-actin, red), and DAPI (nuclei, blue). E denotes the epithelial cells, and images of the fiber cells were taken in sequence along similar areas of the lens. EphA2-pS898 staining signals were enriched in mature lens fiber cells (asterisks), and these signals appear to be all around the cell membrane without preference between the broad and short sides of fibers. The staining signal was comparable between control and *ephrin-A5^-/-^* lenses. Scale bar, 20 μm.

**Figure 10:**
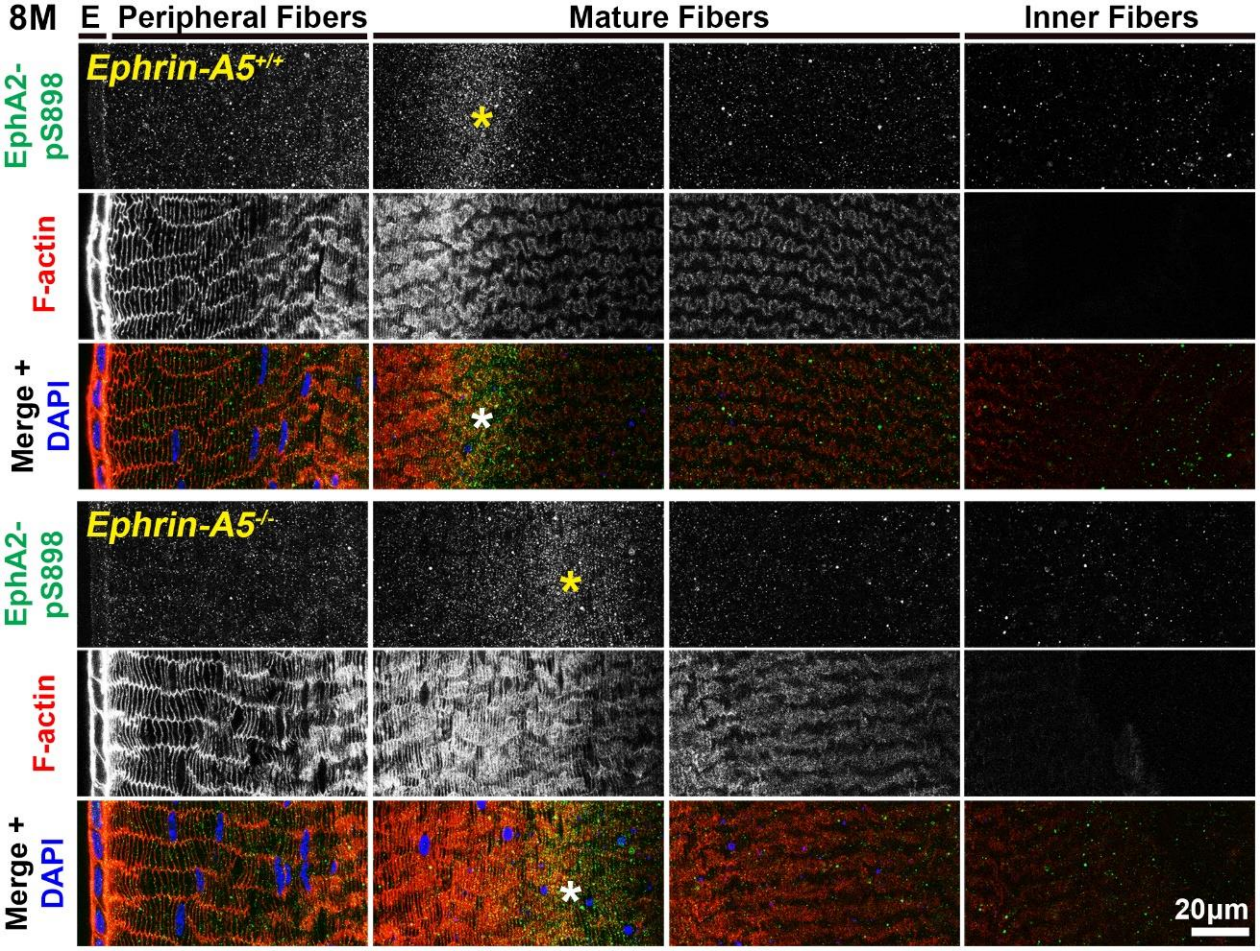
**In lens fiber cells, EphA2-pS898 signal is localized to the cell membrane of mature fibers.** Lens sections in the cross orientation from 8-month-old control and *ephrin-A5^-/-^* mice were stained with EphA2-pS898 (green) antibody, phalloidin (F-actin, red), and DAPI (nuclei, blue). EphA2-pS898 signal was present in mature fiber cells (asterisks). The signal appeared cytosolic and did not have obvious membrane localization. The staining signals were comparable between control and *ephrin-A5^-/-^* sections. Scale bar, 20 μm.

In sections from 6-week-old, 4-month-old, and 8-month-old *ephrin-A5^+/+^* and *ephrin-A5^-/-^* mice, high magnification images of lens epithelial cells in longitudinal sections revealed very low EphA2-pS898 signal in anterior epithelial cells, and there was an increase in fluorescence signals in equatorial epithelial cells (Figure 8 and Supplementary Figures 11, 12, arrows) with a focal increase at the lens equator. While the staining signal did appear to be near the cell membrane in some epithelial cells, there was no obvious membrane enrichment, and most of the signal appeared to be cytoplasmic. Cross orientation section staining from 6-week-old and 4-month-old *ephrin-A5^+/+^* and *ephrin-A5^-/-^* mice to examine fiber cells showed that EphA2-pS898 staining was weakly present in peripheral fibers and obvious in mature fiber cells (Figures 9 and Supplementary Figure 13, asterisks). In the peripheral fibers, the staining signal appear to be punctate and at or near the cell membrane while in the mature fibers, the staining signal is evenly distributed around the membrane of mature fiber cells without preference for either of the broad or short sides. In the inner fiber cells, the staining signal appears punctate and cytoplasmic. There were no significant differences from EphA2-pS898 staining signals between *ephrin-A5^+/+^* and *ephrin-A5^-/-^* sections. Interestingly, in sections from 8-month-old *ephrin-A5^+/+^* and *ephrin-A5^-/-^* mice, we observed that EphA2-pS898 signals in peripheral and mature fiber cells appeared more cytosolic (Figure 10, asterisks) without obvious enrichment at the cell membrane. These results indicate that non-canonical EphA2 signaling can be active in a physiological tissue, and the data further suggest that loss of ephrin-A5 does not significantly affect the levels or localization of non-canonical EphA2 signaling, thus eliminating the possibility that EMT in the *ephrin-A5^-/-^* lenses is caused by abnormal EphA2 signaling.

## DISCUSSION

**Figure 11:**
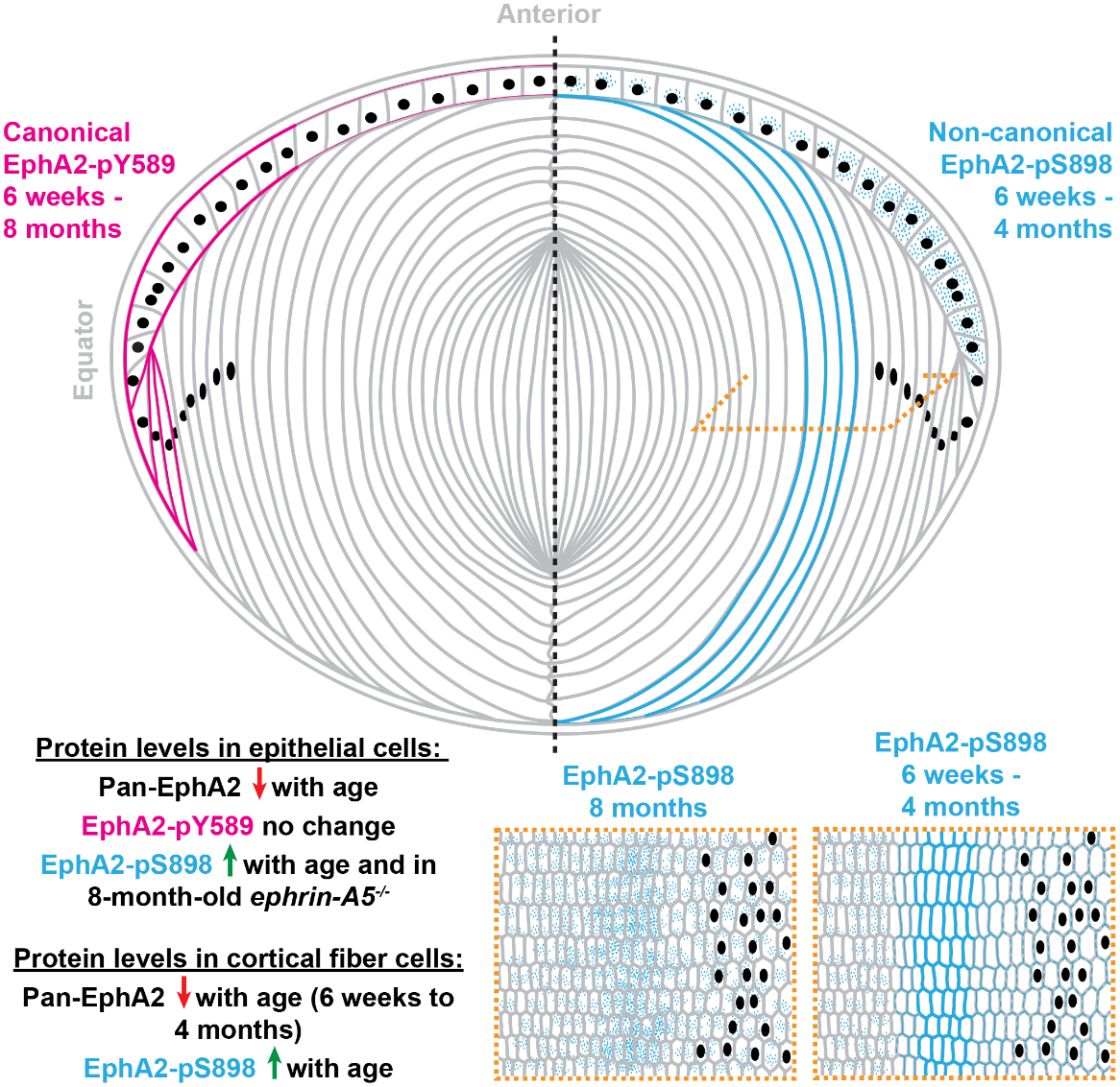
**A summary of the localization and protein levels of canonically active EphA2-pY589 and non-canonically active EphA2-pS898 in control and *ephrin-A5^-/-^* lenses from 6-week-old, 4-month-old, and 8-month-old mice.** Canonical ligand-mediated EphA2-pY589 is only found in lens epithelial cells, and protein levels do not change with age. Non-canonical ligand-independent activation of EphA2-pS898 is found in epithelial cells and mature fiber cells in the lens cortex. The levels of EphA2-pS898 increase with age, and in *ephrin-A5^-/-^* epithelial cells, there is an increase compared to the control in 8-month-old mice. Cartoon not drawn to scale. Modified from [38].

Here, we demonstrated the presence and location of canonical and non-canonical EphA2 signaling within the eye lens (Figure 11). While previous work assumed that EphA2 signals canonically by binding to a ligand in the lens, our work is the first direct evidence that EphA2 is activated by a ligand, and that this bidirectional EphA2 signaling is restricted to epithelial cells, especially at the lens equator. These data further support the role of bidirectional EphA2 signaling in lens epithelial cells to regulate hexagonal cell shape and the formation of organized rows of epithelial and fiber cells [41]. Our Western blots show that the amount of total EphA2 receptor significantly decreased with age in both epithelial and cortical fiber cell fractions. Despite this decrease in protein level, the level of canonical EphA2 activation (pY589) remains steady with age in both control and *ephrin-A5^-/-^* lens epithelial cells. Decreased EphA2 protein levels do not dampen this ligand-mediated activation of the receptor. The lack of obvious changes in canonical EphA2 activation in *ephrin-A5^-/-^* lenses indicates that ephrin-A5 is unlikely to be an exclusive binding partner for EphA2 in the lens, as we previously hypothesized [38, 75].

The presence of non-canonical EphA2 activation in wild-type control lenses is the first demonstration of this signaling in a normal physiological tissue and cell. Non-canonical EphA2 signaling has formerly only been studied in the context of tumor metastasis, carcinogenesis [16, 52, 53, 56, 62, 63, 65–67], and EMT [4, 53, 70]. Being that this is the first time non-canonical EphA2 signaling has been identified in a physiological tissue, the characterization of its activation and localization with age is an important first step to revealing the potential physiological function of ligand-independent EphA2 signaling. The activation of non-canonical EphA2 signaling (pS898) increased with age in control and *ephrin-A5^-/-^* lens epithelial and fiber cells. This increase occurred when there was a decrease in the amount of the pan-EphA2 protein in the lens, making these increases even more significant. Since there are no other reports of physiological activation of EphA2 non-canonical signaling, it is unclear why there is an increase in EphA2 non-canonical signaling with age and whether this increase supports a normal age-related physiological process.

### Non-canonical EphA2 activation in *ephrin-A5^-/-^* lenses

Curiously, in lens epithelial cells from 8-month-old *ephrin-A5^-/-^* mice, there is a large increase in EphA2-pS898 protein compared to the age-matched control samples. We specifically selected *ephrin-A5^-/-^* lenses without obvious anterior epithelial cell EMT and anterior cataracts for our experiments, and thus, this increase is due to changes without the involvement of cells undergoing EMT. We also performed immunostaining in *ephrin-A5^-/-^* lens sections with anterior cataracts that displayed overgrowth of the anterior epithelial cells, and those abnormal epithelial cells do not have staining for non-canonically active EphA2-pS898 (data not shown). The anterior cataracts in *ephrin-A5^-/-^* lenses occur by postnatal day 21 [38, 39] while the rise in EphA2-pS898 non-canonical signaling in *ephrin-A5^-/-^* lenses is only evident in middle-aged 8-month-old KO mice. Thus, it is unlikely that the significant increase in EphA2-pS898 level in lens epithelial cells from 8-month-old *ephrin-A5^-/-^* mice activates EMT in anterior epithelial cells to form cataracts. This evidence further suggests that EphA2 and ephrin-A5 are not an exclusive receptor-ligand pair in the lens.

### Localization of total and activated EphA2 in lens epithelial and fiber cells

There is a gradient of staining signal intensity for pan-EphA2, EphA2-pY589, and EphA2-pS898 at the cell membranes of lens anterior epithelial cells, equatorial epithelial cells, and fiber cells from control and *ephrin-A5^-/-^* lens sections. There is weak EphA2 staining of the anterior epithelial cells compared to equatorial epithelial cells, and there is much stronger staining signal at fiber cell membranes. Canonical signaling localized exclusively to the epithelial cells in both control and *ephrin-A5^-/-^* lenses, with almost no signal detected in the fiber cells. There is a gradient of EphA2-pY589 staining signal with weak staining in anterior lens epithelial cells compared to equatorial epithelial cells. Non-canonical EphA2-pS898 staining signal was present in equatorial epithelial cells and in fiber cells with increased fluorescence within the mature fiber cells. Localization of these staining patterns remained similar with age and genotype indicating that age-related or genotype-related protein level changes have no effect on the cellular localization. The exception to this was the EphA2-pS898 staining pattern in middle-aged control and *ephrin-A5^-/-^* lenses, which we discuss below.

In the anterior epithelial cells, most of the pan-EphA2 staining is concentrated at the apical membrane where the epithelial cells would contact the apical tips of elongating lens fiber cells. This is consistent with our previous report of pan-EphA2 staining in anterior lens epithelial cell flat mount samples [39]. Interestingly, staining for canonically active EphA2-pY589 localizes to both the apical and basal sides of the anterior epithelial cells. This was not expected because staining for pan-EphA2 is not obvious on the basal side of anterior lens epithelial cells. One possible reason for this discrepancy is that the pan-EphA2 staining signal is very strong in lens fibers, and the relatively weaker signal at the basal surface of lens epithelial cells is not visible in these images nor our previous flat mounts because the intensity setting for image collection was determined based on the strong staining signal in fiber cells. In equatorial epithelial cells, we observe pan-EphA2 and canonical EphA2-pY589 staining on both the apical and basal surfaces of the cells. The staining for pan-EphA2 on the basal surface of equatorial epithelial cells is consistent with our previous flat mount data [41].

In contrast, while non-canonical EphA2-pS898 staining signal was present in equatorial epithelial cells, there was no enrichment at the cell membrane, and most of the staining signal appeared in the cytoplasm. It is possible that non-canonical activation the EphA2 leads to rapid receptor internalization. HeLa cells, human breast adenocarcinoma (MDA-MB-231) cells, human lung adenocarcinoma and squamous cell carcinoma biopsy samples, and human vascular smooth muscle cells stained for EphA2-pS897 also revealed a diffuse intracellular pattern [62, 76]. The exclusive activation of canonical EphA2 signaling at epithelial cell membranes, especially at the lens equator, suggests that ligand-mediated EphA2 activity is needed for normal patterning of equatorial epithelial cells. Canonical EphA2 activation is known to signal through Src kinase to affect the actin cytoskeleton [41, 42, 77–79]. In *EphA2^-/-^* and *Src^-/-^* lenses, equatorial epithelial cells fail to properly organize into neat rows of hexagon-shaped cells (Supplementary Figure 2) [41]. Further detailed studies of the localization of canonically and non-canonically active EphA2 in lens epithelial cells using flat mounts is required to better understand the subcellular localization of these phosphorylated forms for EphA2.

In lens fiber cells, pan-EphA2 is enriched along the short sides of mature fiber cells while EphA2-pS898 signal is spread along the entire mature fiber cell membrane. Interestingly, in 8-month-old control and *ephrin-A5^-/-^* mice, the non-canonical EphA2-pS898 signal appeared to be localized more in the mature fiber cell cytoplasm than the membrane, which is an age-related change in protein localization. It is not clear whether the change in the EphA2-pS898 localization may be correlated with cleavage or post-translational modifications that can be seen in the Western blots. Lens fibers cells have very complex morphology that cannot be fully visualized in tissue sections [80, 81], and future immunostaining studies on single lens fiber cells is required to elucidate the subcellular localization of EphA2-pS898 activation in mature lens fiber cells.

### Possible roles of non-canonical EphA2 signaling in lens fiber cell membrane interdigitations and maturation

While the role of non-canonical EphA2 signaling in the normal lens remains unclear, cancerous cells and tissues and immortalized cells with increased non-canonical EphA2 signaling are known to display several phenotypic changes ranging from increased cell motility and invasion [52, 62, 78], resistance to apoptosis [82], promotion of angiogenesis and neovascularization [83–85], and fibrotic tissue responses [9, 76]. Mature lens fiber cells undergo continuous changes in cytoskeletal structure and cell membrane during differentiation with formation of different types of complex membrane interdigitations and protrusions [43, 80, 81, 86]. Though these complex cell interdigitations have been well-described by electron microscopy studies, the mechanisms that influence membrane re-organization during fiber cell differentiation and maturation remain unknown. While different from traditional cell migration, fiber cell membranes extend protrusions and create elaborate cell-cell interlocking features that mimic protrusions from migrating cells. Our previous studies of *EphA2^-/-^* lenses showed distinct changes in inner and perinuclear fiber cell morphology [43]. Scanning electron microscopy studies revealed abnormal cell-cell interdigitations and membrane morphology in a region of the lens that is slightly deeper than where the non-canonical EphA2 signaling is active in mature fiber cells (Supplementary Figure 2). It is possible that non-canonical EphA2 signaling plays a role in the rearrangement of fiber cell membranes in the inner regions of the lens.

EphA2 can be phosphorylated by different AGC protein kinases (Akt, RSK, PKA) resulting in different downstream non-canonical signaling changes [52, 56, 62]. Activation of EphA2 by Akt promotes changes in cytoskeletal elements, through defining the leading edge of migrating cells [52] and enhancing cell motility. Downstream effectors of Akt, like mTORC1, MEK-ERK, and Src-ERK, have been associated with cholangiocarcinoma cells [87], induction of stemness markers [88], and amoeboid motility [89]. Many malignant tumors exhibit cell motility, EMT, and drug resistance because of constitutive non-canonical activation of EphA2 through the MEK-ERK pathway upon growth factor stimulation [9]. Previous studies have suggested that Akt phosphorylation at serine residue 897 is a major mechanism of cell migration in HEK 293 cells and a variety of cancer cells [52]. Known knockouts of elements upstream of Akt display distinct lens phenotypes. PTEN knockouts cause an increase in Akt signaling and lead to lens rupture and cataracts, whereas partial PI3K knockouts lead to reduced growth and equatorial epithelial proliferation [4]. PKA activation has been shown to facilitate the formation of connexin channels within fiber cells, decreasing reactive oxygen species, and alleviating cataract phenotypes [90]. Previous studies have also shown that increased cAMP promotes PKA activity, which results in phosphorylation of EphA2 at serine residue 897, as well as other serine and threonine residues causing a phosphorylation hot spot [56]. These hotspots were enriched at leading edges of migrating cells and are believed to promote malignancy and motility [52, 91]. The PKA-dependent activation of non-canonical EphA2 signaling has further been classified in aggressive pancreatic and prostate cancers [56]. To further determine the pathogenesis of age-related cataracts, future experiments will need to be done to determine the expression levels of activated Akt, RSK and PKA to determine if there are any age-related or genotype-related expression changes and whether there is any link between the pathological and physiological functions of non-canonical EphA2 signaling.

The area of intense EphA2-pS898 staining in mature lens fibers occurs just within the organelle-free zone of the lens after fiber cells remove all cellular organelles. From our data in this study and previous work, we have not observed any obvious changes in the denucleation process in *EphA2^-/-^* lenses. We have not examined the loss of other cellular organelles in *EphA2^-/-^* lenses. Whether non-canonical EphA2 signaling is required for the formation of the organelle-free zone remains to be studied.

### Cataract-causing mutations in human EphA2

Many human mutations in EphA2 are associated with age-related and congenital cataracts [16]. The mechanisms for these variable cataracts remain unknown, but this new data about the two modes of EphA2 activation in the lens can be useful to inform possible changes due to known mutations. Protein models can also be a useful tool in understanding how mutations affect the structural properties of the EphA2. Known cataract causing EphA2 protein sequences can be modeled alongside mutations in residues in close proximity to serine residue 897 or tyrosine residue 588 to observe if there are any significant changes to protein folding. Many human cataracts are reported in the lens cortex where there is increased non-canonical EphA2 activation with age. Future protein modeling work may reveal insights into whether mutations can affect canonical or non-canonical EphA2 phosphorylation, causing cataracts in different regions of the lens.

### Concluding thoughts

The lens is a unique tissue to study the aging of cells since it contains cells that were made during embryonic development that are chronologically old and also newly made cells at the cortex that are still being made in an aged organism. Maintenance of the nuclear fiber cells for an entire lifetime to resist degradation, degeneration, and disease is remarkable, and the mechanisms of normal versus pathological lens aging remain an active area of research.

Here, we report that canonical ligand-mediated EphA2 activation is restricted to the lens epithelial cells and show the first evidence of physiological non-canonical EphA2 activity in a normal tissue. The lens, unexpectedly, utilizes both canonical and non-canonical EphA2 signaling in different cell compartments. Canonical EphA2 signaling is likely important in equatorial lens epithelial cells and may be linked to cell shape changes required for highly ordered packing of the cells to minimize light scattering. Non-canonical EphA2 signaling may also be important in equatorial lens epithelial cells, but the function is unknown due to the mostly cytosolic localization of the activated protein. In lens fibers cells, only non-canonical EphA2 activation is present, and this signaling is enriched in a subset of mature fiber cells undergoing membrane shape changes. We hypothesize that non-canonical EphA2 signaling is important for transformation of complex interdigitations in mature fiber cells.

## MATERIALS AND METHODS

### Mice

*EphA2*^-/-^ mice were acquired from The Jackson Laboratory (strain #: 006028), and e*phrin-A5^-/-^* mice [50] were a generous gift from Dr. David A. Feldheim (University of California, Santa Cruz, CA, USA). All mice were maintained in C57BL/6J background with wild-type Bfsp2 (CP49) genes [39]. Sporadic mutations in Bfsp2 are associated with several mouse strains and can influence lens fiber cell cytoskeleton and produce cataracts not associated with EphA2 signaling pathways or aging [80, 92–95]. Genotyping was completed using automated qPCR on toe and/or tail samples collected between postnatal days 5-7 (Transnetyx). Age-matched littermates from heterozygous mating pairs were used for experiments to prevent differences due to genetic background drift. Approximately equal numbers of male and female mice were used in our experiments.

### Lens protein extraction and JESS capillary-based immunoassay

Fresh enucleated eyes were dissected in 1X phosphate buffered saline (PBS, 14190-144, Gibco) in a dissection plate as previously described [96]. Separation of the lens epithelial layer, cortical fiber cells, and nuclear fiber cell masses was also performed as previously described [97]. Samples were collected from 6-week-old, 4-month-old, and 8-month-old *EphA2^+/+^*, *EphA2^-/-^*, *ephrin-A5^+/+^*, and *ephrin-A5^-/-^* mice. At least three different mice per genotype per age were used to make biological replicates. For *ephrin-A5^-/-^* mice, samples were only collected from pairs of eyes without obvious anterior cataracts because it would not be possible to separate the signaling that is altered by the loss of ephrin-A5 from changes that occurred after EMT is initiated in the anterior epithelial cells.

Briefly, the lens epithelium attached to the capsule was separated from the fiber cell mass, and the capsule from two eyes from the same mouse was placed into a 1.5 mL microcentrifuge tube filled with 14.75 μl of ice-cold lens homogenization buffer [20mM Tris (pH 7.4 at 4° C), 100mM sodium chloride, 1mM magnesium chloride, 2mM ethylene glycol tetraacetic acid, 10mM sodium fluoride, 1mM dithiothreitol (DTT), 1X Pierce phosphatase inhibitor (A32957, Thermo Fisher Scientific), 1:100 protease inhibitor cocktail (P8340, Sigma-Aldrich)] and 1.25 μL of 2X sample buffer [0.21M Tris-HCl (pH 6.8), 2.86 mM ethylenediamine tetra-acetic ETDA, 21% sucrose, 6.67% SDS, and 0.3M DTT in ddH2O]. DTT, Pierce phosphatase inhibitor, and the protease inhibitor cocktail were added to the homogenization buffer on the day of sample collection. Lens epithelial cell samples were then vortexed briefly with two short pulses followed by brief centrifugation at room temperature at 2,000x *g* for 15 seconds. To separate the cortical and nuclear fiber cells, the fiber cell mass was added to a 1.5 mL microcentrifuge tube with 250 μL of 1:1 ice-cold lens homogenization buffer and 2X sample buffer and vortexed in 30-second bursts over 2-4 minutes. Vortexing can be stopped earlier than 4 minutes if the ball of fiber cells does not become smaller with successive vortexing. The remaining ball of fiber cells are the dense and compact lens nuclear fiber cells, which were removed with clean tweezers from the cortical fiber cell lysate and discarded. Sonication (QSonica, Q55-110, with 5/64” probe, 4423, at an amplitude of 15) of lens cell fractions was performed using cycles of 3 seconds of sonication and 10 seconds of cooling on ice. Epithelial cells received one cycle of sonication while fiber cells received three cycles. After sonication, the lens lysate protein concentration was measured using the Bradford assay (5000205, BioRad) per the manufacturer’s instructions.

Western blot analysis was performed using the JESS capillary electrophoresis machine (ProteinSimple) to minimize the amount of sample needed for measurements. Equal amounts of control and KO proteins for lens epithelial or cortical fiber cell fractions were loaded into the JESS 12-230kDa separation module kit as per manufacturer instructions. Previously calculated protein and antibody concentration titrations were used to determine the range for antibody saturation and optimum concentrations of protein extracts. Detection of protein was performed by loading lens epithelial cells (~0.4 μg/μL) and cortical fiber cells (3 μg/μL) with 1:50 of a rabbit anti-EphA2 antibody (6997, Cell Signaling Technology), 1:50 of a rabbit anti-EphA2-pS897 antibody (6347, Cell Signaling Technology) or 1:50 of a rabbit-EphA2-pY588 antibody (12677, Cell Signaling Technology). While both antibodies against the phosphorylated forms of EphA2 were made to the human protein sequence, there is 100% homology between the human and mouse EphA2 protein sequence in the regions recognized by the antibodies. Antibody specificity was verified by lack of signal in *EphA2^-/-^* samples (Supplementary Figures 3, 4). Target protein levels were normalized to the total protein signal from each capillary measured using the Total Protein detection kit from ProteinSimple. Calculations for average and standard deviation were completed in Excel and plotted in GraphPad 9. ANOVA (1-way) was used to determine statistical relevance, p < 0.05, between control and KO samples from different age groups.

### Immunostaining

Frozen lens tissue sections were collected and prepared for fixation as previously described [81, 98]. To summarize, eyes from 6-week-, 4-month-, and 8-month-old *EphA2^+/+^*, *EphA2^-/-^*, *ephrin-A5^+/+^*, and *ephrin-A5^-/-^* mice were collected and fixed with freshly made ice-cold 1% paraformaldehyde (PFA, 15710, Electron Microscopy Sciences). For *ephrin-A5^-/-^* eyes, sections from lenses with obvious anterior cataracts were excluded. A small incision was made into the corneal-scleral junction, allowing the PFA fixative to better penetrate the globe. The eyes were cryoprotected in a 30% sucrose solution before being frozen in OCT medium, oriented in the cross or longitudinal orientation. Twelve-micron thick slices were collected using a Leica CM1860 cryostat. Sections were stored at -20° C until staining.

Frozen tissue sections were washed with 1X PBS + 0.1% Triton X-100 (PTX) before being incubated in blocking buffer (5% donkey serum + 0.3% Triton X-100 in 1X PBS). A 1:100 dilution of primary antibody (same antibodies as described for western blots) was applied, and sections were incubated overnight at 4° C. Slides were washed in 1X PTX with light rocking before a 1:200 dilution of donkey anti-rabbit secondary antibody conjugated to Alexa-Fluor-488 (711-545-152, Jackson ImmunoResearch Laboratories) and rhodamine phalloidin (R415, Thermo Fisher Scientific) was applied to the sections and incubated. Stained sections were washed in 1X PTX and then mounted with Vectashield mounting media containing DAPI (H-1200, Vectashield). The edges of the coverslip were sealed with clear nail polish.

Sections were viewed using a Zeiss LSM800 laser confocal microscope with 20X [air, numerical aperture (NA) = 0.8] and 63X (oil, NA = 1.4) objectives, for a total magnification of 200X and 630X, respectively. Images were captured using Zen 2.3 SPI software at room temperature on the same laser and gain intensities. Comparable equatorial lens sections were selected from judging thickness of the epithelial monolayer, and presence of nuclei in the fiber cells. Staining was repeated on at least 3 biological replicates of each genotype and age. Representative data are shown.

## Supplementary Material

Supplementary Figures

## References

[1] Pasquale EB. Eph receptors and ephrins in cancer: bidirectional signalling and beyond. Nat Rev Cancer. 2010; 10:165–80. 10.1038/nrc2806 20179713 PMC2921274

[2] Darling TK, Lamb TJ. Emerging Roles for Eph Receptors and Ephrin Ligands in Immunity. Front Immunol. 2019; 10:1473. 10.3389/fimmu.2019.01473 31333644 PMC6620610

[3] Pitulescu ME, Adams RH. Eph/ephrin molecules--a hub for signaling and endocytosis. Genes Dev. 2010; 24:2480–92. 10.1101/gad.1973910 21078817 PMC2975924

[4] Zhang C, Smalley I, Emmons MF, Sharma R, Izumi V, Messina J, Koomen JM, Pasquale EB, Forsyth PA, Smalley KS. Noncanonical EphA2 Signaling Is a Driver of Tumor-Endothelial Cell Interactions and Metastatic Dissemination in BRAF Inhibitor-Resistant Melanoma. J Invest Dermatol. 2021; 141:840–51.e4. 10.1016/j.jid.2020.08.012 32890629 PMC7921215

[5] Pasquale EB. Eph receptor signalling casts a wide net on cell behaviour. Nat Rev Mol Cell Biol. 2005; 6:462–75. 10.1038/nrm1662 15928710

[6] Wilson K, Shiuan E, Brantley-Sieders DM. Oncogenic functions and therapeutic targeting of EphA2 in cancer. Oncogene. 2021; 40:2483–95. 10.1038/s41388-021-01714-8 33686241 PMC8035212

[7] Brannan JM, Sen B, Saigal B, Prudkin L, Behrens C, Solis L, Dong W, Bekele BN, Wistuba I, Johnson FM. EphA2 in the early pathogenesis and progression of non-small cell lung cancer. Cancer Prev Res (Phila). 2009; 2:1039–49. 10.1158/1940-6207.CAPR-09-0212 19934338

[8] Larsen AB, Stockhausen MT, Poulsen HS. Cell adhesion and EGFR activation regulate EphA2 expression in cancer. Cell Signal. 2010; 22:636–44. 10.1016/j.cellsig.2009.11.018 19948216

[9] Zhou Y, Oki R, Tanaka A, Song L, Takashima A, Hamada N, Yokoyama S, Yano S, Sakurai H. Cellular stress induces non-canonical activation of the receptor tyrosine kinase EphA2 through the p38-MK2-RSK signaling pathway. J Biol Chem. 2023; 299:104699. 10.1016/j.jbc.2023.104699 37059179 PMC10196987

[10] Canovas B, Nebreda AR. Diversity and versatility of p38 kinase signalling in health and disease. Nat Rev Mol Cell Biol. 2021; 22:346–66. 10.1038/s41580-020-00322-w 33504982 PMC7838852

[11] Igea A, Nebreda AR. The Stress Kinase p38α as a Target for Cancer Therapy. Cancer Res. 2015; 75:3997–4002. 10.1158/0008-5472.CAN-15-0173 26377941

[12] Liang LY, Patel O, Janes PW, Murphy JM, Lucet IS. Eph receptor signalling: from catalytic to non-catalytic functions. Oncogene. 2019; 38:6567–84. 10.1038/s41388-019-0931-2 31406248

[13] Matsuoka H, Obama H, Kelly ML, Matsui T, Nakamoto M. Biphasic functions of the kinase-defective Ephb6 receptor in cell adhesion and migration. J Biol Chem. 2005; 280:29355–63. 10.1074/jbc.M500010200 15955811

[14] Pasquale EB. Eph-ephrin bidirectional signaling in physiology and disease. Cell. 2008; 133:38–52. 10.1016/j.cell.2008.03.011 18394988

[15] Jørgensen C, Sherman A, Chen GI, Pasculescu A, Poliakov A, Hsiung M, Larsen B, Wilkinson DG, Linding R, Pawson T. Cell-specific information processing in segregating populations of Eph receptor ephrin-expressing cells. Science. 2009; 326:1502–9. 10.1126/science.1176615 20007894

[16] Murugan S, Cheng C. Roles of Eph-Ephrin Signaling in the Eye Lens Cataractogenesis, Biomechanics, and Homeostasis. Front Cell Dev Biol. 2022; 10:852236. 10.3389/fcell.2022.852236 35295853 PMC8918484

[17] Noren NK, Pasquale EB. Eph receptor-ephrin bidirectional signals that target Ras and Rho proteins. Cell Signal. 2004; 16:655–66. 10.1016/j.cellsig.2003.10.006 15093606

[18] Barquilla A, Pasquale EB. Eph receptors and ephrins: therapeutic opportunities. Annu Rev Pharmacol Toxicol. 2015; 55:465–87. 10.1146/annurev-pharmtox-011112-140226 25292427 PMC4388660

[19] Pasquale EB. Eph-ephrin promiscuity is now crystal clear. Nat Neurosci. 2004; 7:417–8. 10.1038/nn0504-417 15114347

[20] Lisabeth EM, Falivelli G, Pasquale EB. Eph receptor signaling and ephrins. Cold Spring Harb Perspect Biol. 2013; 5:a009159. 10.1101/cshperspect.a009159 24003208 PMC3753714

[21] Noberini R, Rubio de la Torre E, Pasquale EB. Profiling Eph receptor expression in cells and tissues: a targeted mass spectrometry approach. Cell Adh Migr. 2012; 6:102–12. 10.4161/cam.19620 22568954 PMC3499309

[22] Himanen JP, Saha N, Nikolov DB. Cell-cell signaling via Eph receptors and ephrins. Curr Opin Cell Biol. 2007; 19:534–42. 10.1016/j.ceb.2007.08.004 17928214 PMC3327877

[23] Shiels A, Hejtmancik JF. Mutations and mechanisms in congenital and age-related cataracts. Exp Eye Res. 2017; 156:95–102. 10.1016/j.exer.2016.06.011 27334249 PMC5538314

[24] Jun G, Guo H, Klein BE, Klein R, Wang JJ, Mitchell P, Miao H, Lee KE, Joshi T, Buck M, Chugha P, Bardenstein D, Klein AP, et al. EPHA2 is associated with age-related cortical cataract in mice and humans. PLoS Genet. 2009; 5:e1000584. 10.1371/journal.pgen.1000584 19649315 PMC2712078

[25] Zhang T, Hua R, Xiao W, Burdon KP, Bhattacharya SS, Craig JE, Shang D, Zhao X, Mackey DA, Moore AT, Luo Y, Zhang J, Zhang X. Mutations of the EPHA2 receptor tyrosine kinase gene cause autosomal dominant congenital cataract. Hum Mutat. 2009; 30:E603–11. 10.1002/humu.20995 19306328

[26] Kaul H, Riazuddin SA, Shahid M, Kousar S, Butt NH, Zafar AU, Khan SN, Husnain T, Akram J, Hejtmancik JF, Riazuddin S. Autosomal recessive congenital cataract linked to EPHA2 in a consanguineous Pakistani family. Mol Vis. 2010; 16:511–7. 20361013 PMC2846848

[27] Lin Q, Zhou N, Zhang N, Qi Y. Mutational screening of EFNA5 in Chinese age-related cataract patients. Ophthalmic Res. 2014; 52:124–9. 10.1159/000363139 25300504

[28] Tan W, Hou S, Jiang Z, Hu Z, Yang P, Ye J. Association of EPHA2 polymorphisms and age-related cortical cataract in a Han Chinese population. Mol Vis. 2011; 17:1553–8. 21686326 PMC3115745

[29] Park JE, Son AI, Hua R, Wang L, Zhang X, Zhou R. Human cataract mutations in EPHA2 SAM domain alter receptor stability and function. PLoS One. 2012; 7:e36564. 10.1371/journal.pone.0036564 22570727 PMC3343017

[30] Dave A, Laurie K, Staffieri SE, Taranath D, Mackey DA, Mitchell P, Wang JJ, Craig JE, Burdon KP, Sharma S. Mutations in the EPHA2 gene are a major contributor to inherited cataracts in South-Eastern Australia. PLoS One. 2013; 8:e72518. 10.1371/journal.pone.0072518 24014202 PMC3754966

[31] Li D, Wang S, Ye H, Tang Y, Qiu X, Fan Q, Rong X, Liu X, Chen Y, Yang J, Lu Y. Distribution of gene mutations in sporadic congenital cataract in a Han Chinese population. Mol Vis. 2016; 22:589–98. 27307692 PMC4896834

[32] Berry V, Pontikos N, Albarca-Aguilera M, Plagnol V, Massouras A, Prescott D, Moore AT, Arno G, Cheetham ME, Michaelides M. A recurrent splice-site mutation in EPHA2 causing congenital posterior nuclear cataract. Ophthalmic Genet. 2018; 39:236–41. 10.1080/13816810.2017.1381977 29039721

[33] Zhai Y, Zhu S, Li J, Yao K. A Novel Human Congenital Cataract Mutation in EPHA2 Kinase Domain (p.G668D) Alters Receptor Stability and Function. Invest Ophthalmol Vis Sci. 2019; 60:4717–26. 10.1167/iovs.19-27370 31725171

[34] Sundaresan P, Ravindran RD, Vashist P, Shanker A, Nitsch D, Talwar B, Maraini G, Camparini M, Nonyane BA, Smeeth L, Chakravarthy U, Hejtmancik JF, Fletcher AE. EPHA2 polymorphisms and age-related cataract in India. PLoS One. 2012; 7:e33001. 10.1371/journal.pone.0033001 22412971 PMC3297613

[35] Lovicu FJ, Robinson ML. Development of the Ocular Lens: Cambridge University Press). 2011.

[36] Bassnett S. Lens organelle degradation. Exp Eye Res. 2002; 74:1–6. 10.1006/exer.2001.1111 11878813

[37] Son AI, Cooper MA, Sheleg M, Sun Y, Kleiman NJ, Zhou R. Further analysis of the lens of ephrin-A5-/- mice: development of postnatal defects. Mol Vis. 2013; 19:254–66. 23401654 PMC3566898

[38] Cheng C, Fowler VM, Gong X. EphA2 and ephrin-A5 are not a receptor-ligand pair in the ocular lens. Exp Eye Res. 2017; 162:9–17. 10.1016/j.exer.2017.06.016 28648759 PMC5554726

[39] Cheng C, Gong X. Diverse roles of Eph/ephrin signaling in the mouse lens. PLoS One. 2011; 6:e28147. 10.1371/journal.pone.0028147 22140528 PMC3226676

[40] Zhou Y, Shiels A. Epha2 and Efna5 participate in lens cell pattern-formation. Differentiation. 2018; 102:1–9. 10.1016/j.diff.2018.05.002 29800803 PMC6287607

[41] Cheng C, Ansari MM, Cooper JA, Gong X. EphA2 and Src regulate equatorial cell morphogenesis during lens development. Development. 2013; 140:4237–45. 10.1242/dev.100727 24026120 PMC3787762

[42] Zhou Y, Bennett TM, Ruzycki PA, Shiels A. Mutation of the EPHA2 Tyrosine-Kinase Domain Dysregulates Cell Pattern Formation and Cytoskeletal Gene Expression in the Lens. Cells. 2021; 10:2606. 10.3390/cells10102606 34685586 PMC8534143

[43] Cheng C, Wang K, Hoshino M, Uesugi K, Yagi N, Pierscionek B. EphA2 Affects Development of the Eye Lens Nucleus and the Gradient of Refractive Index. Invest Ophthalmol Vis Sci. 2022; 63:2. 10.1167/iovs.63.1.2 34978559 PMC8742528

[44] Carter N, Nakamoto T, Hirai H, Hunter T. EphrinA1-induced cytoskeletal re-organization requires FAK and p130(cas). Nat Cell Biol. 2002; 4:565–73. 10.1038/ncb823 12134157

[45] Yang NY, Pasquale EB, Owen LB, Ethell IM. The EphB4 receptor-tyrosine kinase promotes the migration of melanoma cells through Rho-mediated actin cytoskeleton reorganization. J Biol Chem. 2006; 281:32574–86. 10.1074/jbc.M604338200 16950769

[46] Davy A, Gale NW, Murray EW, Klinghoffer RA, Soriano P, Feuerstein C, Robbins SM. Compartmentalized signaling by GPI-anchored ephrin-A5 requires the Fyn tyrosine kinase to regulate cellular adhesion. Genes Dev. 1999; 13:3125–35. 10.1101/gad.13.23.3125 10601038 PMC317175

[47] Davy A, Robbins SM. Ephrin-A5 modulates cell adhesion and morphology in an integrin-dependent manner. EMBO J. 2000; 19:5396–405. 10.1093/emboj/19.20.5396 11032807 PMC314006

[48] Wang HU, Chen ZF, Anderson DJ. Molecular distinction and angiogenic interaction between embryonic arteries and veins revealed by ephrin-B2 and its receptor Eph-B4. Cell. 1998; 93:741–53. 10.1016/s0092-8674(00)81436-1 9630219

[49] Xu Q, Alldus G, Holder N, Wilkinson DG. Expression of truncated Sek-1 receptor tyrosine kinase disrupts the segmental restriction of gene expression in the Xenopus and zebrafish hindbrain. Development. 1995; 121:4005–16. 10.1242/dev.121.12.4005 8575301

[50] Frisén J, Yates PA, McLaughlin T, Friedman GC, O’Leary DD, Barbacid M. Ephrin-A5 (AL-1/RAGS) is essential for proper retinal axon guidance and topographic mapping in the mammalian visual system. Neuron. 1998; 20:235–43. 10.1016/s0896-6273(00)80452-3 9491985

[51] Marler KJ, Becker-Barroso E, Martínez A, Llovera M, Wentzel C, Poopalasundaram S, Hindges R, Soriano E, Comella J, Drescher U. A TrkB/EphrinA interaction controls retinal axon branching and synaptogenesis. J Neurosci. 2008; 28:12700–12. 10.1523/JNEUROSCI.1915-08.2008 19036963 PMC3844751

[52] Miao H, Li DQ, Mukherjee A, Guo H, Petty A, Cutter J, Basilion JP, Sedor J, Wu J, Danielpour D, Sloan AE, Cohen ML, Wang B. EphA2 mediates ligand-dependent inhibition and ligand-independent promotion of cell migration and invasion via a reciprocal regulatory loop with Akt. Cancer Cell. 2009; 16:9–20. 10.1016/j.ccr.2009.04.009 19573808 PMC2860958

[53] Lechtenberg BC, Gehring MP, Light TP, Horne CR, Matsumoto MW, Hristova K, Pasquale EB. Regulation of the EphA2 receptor intracellular region by phosphomimetic negative charges in the kinase-SAM linker. Nat Commun. 2021; 12:7047. 10.1038/s41467-021-27343-z 34857764 PMC8639986

[54] Miao H, Wei BR, Peehl DM, Li Q, Alexandrou T, Schelling JR, Rhim JS, Sedor JR, Burnett E, Wang B. Activation of EphA receptor tyrosine kinase inhibits the Ras/MAPK pathway. Nat Cell Biol. 2001; 3:527–30. 10.1038/35074604 11331884

[55] Yang NY, Fernandez C, Richter M, Xiao Z, Valencia F, Tice DA, Pasquale EB. Crosstalk of the EphA2 receptor with a serine/threonine phosphatase suppresses the Akt-mTORC1 pathway in cancer cells. Cell Signal. 2011; 23:201–12. 10.1016/j.cellsig.2010.09.004 20837138 PMC2972709

[56] Barquilla A, Lamberto I, Noberini R, Heynen-Genel S, Brill LM, Pasquale EB. Protein kinase A can block EphA2 receptor-mediated cell repulsion by increasing EphA2 S897 phosphorylation. Mol Biol Cell. 2016; 27:2757–70. 10.1091/mbc.E16-01-0048 27385333 PMC5007095

[57] Bush JO. Cellular and molecular mechanisms of EPH/EPHRIN signaling in evolution and development. Curr Top Dev Biol. 2022; 149:153–201. 10.1016/bs.ctdb.2022.02.005 35606056

[58] Koshikawa N, Hoshino D, Taniguchi H, Minegishi T, Tomari T, Nam SO, Aoki M, Sueta T, Nakagawa T, Miyamoto S, Nabeshima K, Weaver AM, Seiki M. Proteolysis of EphA2 Converts It from a Tumor Suppressor to an Oncoprotein. Cancer Res. 2015; 75:3327–39. 10.1158/0008-5472.CAN-14-2798 26130649 PMC4682662

[59] Park JE, Son AI, Zhou R. Roles of EphA2 in Development and Disease. Genes (Basel). 2013; 4:334–57. 10.3390/genes4030334 24705208 PMC3924825

[60] Singh DR, Ahmed F, Paul MD, Gedam M, Pasquale EB, Hristova K. The SAM domain inhibits EphA2 interactions in the plasma membrane. Biochim Biophys Acta Mol Cell Res. 2017; 1864:31–8. 10.1016/j.bbamcr.2016.10.011 27776928 PMC5148718

[61] Singh DR, Cao Q, King C, Salotto M, Ahmed F, Zhou XY, Pasquale EB, Hristova K. Unliganded EphA3 dimerization promoted by the SAM domain. Biochem J. 2015; 471:101–9. 10.1042/BJ20150433 26232493 PMC4692061

[62] Zhou Y, Yamada N, Tanaka T, Hori T, Yokoyama S, Hayakawa Y, Yano S, Fukuoka J, Koizumi K, Saiki I, Sakurai H. Crucial roles of RSK in cell motility by catalysing serine phosphorylation of EphA2. Nat Commun. 2015; 6:7679. 10.1038/ncomms8679 26158630 PMC4510653

[63] Xiao T, Xiao Y, Wang W, Tang YY, Xiao Z, Su M. Targeting EphA2 in cancer. J Hematol Oncol. 2020; 13:114. 10.1186/s13045-020-00944-9 32811512 PMC7433191

[64] Singh DR, Kanvinde P, King C, Pasquale EB, Hristova K. The EphA2 receptor is activated through induction of distinct, ligand-dependent oligomeric structures. Commun Biol. 2018; 1:15. 10.1038/s42003-018-0017-7 30271902 PMC6123813

[65] Ireton RC, Chen J. EphA2 receptor tyrosine kinase as a promising target for cancer therapeutics. Curr Cancer Drug Targets. 2005; 5:149–57. 10.2174/1568009053765780 15892616

[66] Fang WB, Brantley-Sieders DM, Parker MA, Reith AD, Chen J. A kinase-dependent role for EphA2 receptor in promoting tumor growth and metastasis. Oncogene. 2005; 24:7859–68. 10.1038/sj.onc.1208937 16103880

[67] Zelinski DP, Zantek ND, Stewart JC, Irizarry AR, Kinch MS. EphA2 overexpression causes tumorigenesis of mammary epithelial cells. Cancer Res. 2001; 61:2301–6. 11280802

[68] Wykosky J, Gibo DM, Stanton C, Debinski W. EphA2 as a novel molecular marker and target in glioblastoma multiforme. Mol Cancer Res. 2005; 3:541–51. 10.1158/1541-7786.MCR-05-0056 16254188

[69] Jukonen J, Moyano-Galceran L, Höpfner K, Pietilä EA, Lehtinen L, Huhtinen K, Gucciardo E, Hynninen J, Hietanen S, Grénman S, Ojala PM, Carpén O, Lehti K. Aggressive and recurrent ovarian cancers upregulate ephrinA5, a non-canonical effector of EphA2 signaling duality. Sci Rep. 2021; 11:8856. 10.1038/s41598-021-88382-6 33893375 PMC8065122

[70] Zhou Y, Sakurai H. Emerging and Diverse Functions of the EphA2 Noncanonical Pathway in Cancer Progression. Biol Pharm Bull. 2017; 40:1616–24. 10.1248/bpb.b17-00446 28966234

[71] Lemmon MA, Schlessinger J. Cell signaling by receptor tyrosine kinases. Cell. 2010; 141:1117–34. 10.1016/j.cell.2010.06.011 20602996 PMC2914105

[72] Anderton M, van der Meulen E, Blumenthal MJ, Schäfer G. The Role of the Eph Receptor Family in Tumorigenesis. Cancers (Basel). 2021; 13:206. 10.3390/cancers13020206 33430066 PMC7826860

[73] Cheng C, Parreno J, Nowak RB, Biswas SK, Wang K, Hoshino M, Uesugi K, Yagi N, Moncaster JA, Lo WK, Pierscionek B, Fowler VM. Age-related changes in eye lens biomechanics, morphology, refractive index and transparency. Aging (Albany NY). 2019; 11:12497–531. 10.18632/aging.102584 31844034 PMC6949082

[74] Li D, Han X, Zhao Z, Lu Y, Yang J. Functional analysis of deleterious EPHA2 SNPs in lens epithelial cells. Mol Vis. 2021; 27:384–95. 34220184 PMC8219505

[75] Cheng C. EphA2 and Ephrin-A5 Guide Eye Lens Suture Alignment and Influence Whole Lens Resilience. Invest Ophthalmol Vis Sci. 2021; 62:3. 10.1167/iovs.62.15.3 34854885 PMC8648058

[76] Finney AC, Funk SD, Green JM, Yurdagul A Jr, Rana MA, Pistorius R, Henry M, Yurochko A, Pattillo CB, Traylor JG, Chen J, Woolard MD, Kevil CG, Orr AW. EphA2 Expression Regulates Inflammation and Fibroproliferative Remodeling in Atherosclerosis. Circulation. 2017; 136:566–82. 10.1161/CIRCULATIONAHA.116.026644 28487392 PMC5548618

[77] Baldwin C, Chen ZW, Bedirian A, Yokota N, Nasr SH, Rabb H, Lemay S. Upregulation of EphA2 during *in vivo* and *in vitro* renal ischemia-reperfusion injury: role of Src kinases. Am J Physiol Renal Physiol. 2006; 291:F960–71. 10.1152/ajprenal.00020.2006 16735461

[78] Faoro L, Singleton PA, Cervantes GM, Lennon FE, Choong NW, Kanteti R, Ferguson BD, Husain AN, Tretiakova MS, Ramnath N, Vokes EE, Salgia R. EphA2 mutation in lung squamous cell carcinoma promotes increased cell survival, cell invasion, focal adhesions, and mammalian target of rapamycin activation. J Biol Chem. 2010; 285:18575–85. 10.1074/jbc.M109.075085 20360610 PMC2881783

[79] Parri M, Buricchi F, Giannoni E, Grimaldi G, Mello T, Raugei G, Ramponi G, Chiarugi P. EphrinA1 activates a Src/focal adhesion kinase-mediated motility response leading to rho-dependent actino/myosin contractility. J Biol Chem. 2007; 282:19619–28. 10.1074/jbc.M701319200 17449913

[80] Cheng C, Nowak RB, Amadeo MB, Biswas SK, Lo WK, Fowler VM. Tropomyosin 3.5 protects the F-actin networks required for tissue biomechanical properties. J Cell Sci. 2018; 131:jcs222042. 10.1242/jcs.222042 30333143 PMC6288072

[81] Cheng C, Nowak RB, Biswas SK, Lo WK, FitzGerald PG, Fowler VM. Tropomodulin 1 Regulation of Actin Is Required for the Formation of Large Paddle Protrusions Between Mature Lens Fiber Cells. Invest Ophthalmol Vis Sci. 2016; 57:4084–99. 10.1167/iovs.16-19949 27537257 PMC4986768

[82] Harada K, Hiramoto-Yamaki N, Negishi M, Katoh H. Ephexin4 and EphA2 mediate resistance to anoikis through RhoG and phosphatidylinositol 3-kinase. Exp Cell Res. 2011; 317:1701–13. 10.1016/j.yexcr.2011.05.014 21621533

[83] Zhao P, Jiang D, Huang Y, Chen C. EphA2: A promising therapeutic target in breast cancer. J Genet Genomics. 2021; 48:261–7. 10.1016/j.jgg.2021.02.011 33962882

[84] Baharuddin WN, Yusoff AA, Abdullah JM, Osman ZF, Ahmad F. Roles of EphA2 Receptor in Angiogenesis Signaling Pathway of Glioblastoma Multiforme. Malays J Med Sci. 2018; 25:22–7. 10.21315/mjms2018.25.6.3 30914876 PMC6422564

[85] Han B, Zhang H, Tian R, Liu H, Wang Z, Wang Z, Tian J, Cui Y, Ren S, Zuo X, Tian R, Niu R, Zhang F. Exosomal EPHA2 derived from highly metastatic breast cancer cells promotes angiogenesis by activating the AMPK signaling pathway through Ephrin A1-EPHA2 forward signaling. Theranostics. 2022; 12:4127–46. 10.7150/thno.72404 35673569 PMC9169374

[86] Vu MP, Cheng C. Preparation and Immunofluorescence Staining of Bundles and Single Fiber Cells from the Cortex and Nucleus of the Eye Lens. J Vis Exp. 2023; (196):10.3791/65638. 10.3791/65638 37358269 PMC10729647

[87] Cui XD, Lee MJ, Kim JH, Hao PP, Liu L, Yu GR, Kim DG. Activation of mammalian target of rapamycin complex 1 (mTORC1) and Raf/Pyk2 by growth factor-mediated Eph receptor 2 (EphA2) is required for cholangiocarcinoma growth and metastasis. Hepatology. 2013; 57:2248–60. 10.1002/hep.26253 23315987

[88] Binda E, Visioli A, Giani F, Lamorte G, Copetti M, Pitter KL, Huse JT, Cajola L, Zanetti N, DiMeco F, De Filippis L, Mangiola A, Maira G, et al. The EphA2 receptor drives self-renewal and tumorigenicity in stem-like tumor-propagating cells from human glioblastomas. Cancer Cell. 2012; 22:765–80. 10.1016/j.ccr.2012.11.005 23238013 PMC3922047

[89] Taddei ML, Parri M, Angelucci A, Bianchini F, Marconi C, Giannoni E, Raugei G, Bologna M, Calorini L, Chiarugi P. EphA2 induces metastatic growth regulating amoeboid motility and clonogenic potential in prostate carcinoma cells. Mol Cancer Res. 2011; 9:149–60. 10.1158/1541-7786.MCR-10-0298 21205836

[90] Du Y, Tong Y, Quan Y, Wang G, Cheng H, Gu S, Jiang JX. Protein kinase A activation alleviates cataract formation via increased gap junction intercellular communication. iScience. 2023; 26:106114. 10.1016/j.isci.2023.106114 36852280 PMC9958365

[91] Miao H, Wang B. Eph/ephrin signaling in epithelial development and homeostasis. Int J Biochem Cell Biol. 2009; 41:762–70. 10.1016/j.biocel.2008.07.019 18761422 PMC3108796

[92] Alizadeh A, Clark J, Seeberger T, Hess J, Blankenship T, FitzGerald PG. Characterization of a mutation in the lens-specific CP49 in the 129 strain of mouse. Invest Ophthalmol Vis Sci. 2004; 45:884–91. 10.1167/iovs.03-0677 14985306

[93] Gokhin DS, Nowak RB, Kim NE, Arnett EE, Chen AC, Sah RL, Clark JI, Fowler VM. Tmod1 and CP49 synergize to control the fiber cell geometry, transparency, and mechanical stiffness of the mouse lens. PLoS One. 2012; 7:e48734. 10.1371/journal.pone.0048734 23144950 PMC3492431

[94] Sandilands A, Wang X, Hutcheson AM, James J, Prescott AR, Wegener A, Pekny M, Gong X, Quinlan RA. Bfsp2 mutation found in mouse 129 strains causes the loss of CP49' and induces vimentin-dependent changes in the lens fibre cell cytoskeleton. Exp Eye Res. 2004; 78:875–89. 10.1016/j.exer.2003.09.028 15037121

[95] Simirskii VN, Lee RS, Wawrousek EF, Duncan MK. Inbred FVB/N mice are mutant at the cp49/Bfsp2 locus and lack beaded filament proteins in the lens. Invest Ophthalmol Vis Sci. 2006; 47:4931–4. 10.1167/iovs.06-0423 17065509

[96] Cheng C, Gokhin DS, Nowak RB, Fowler VM. Sequential Application of Glass Coverslips to Assess the Compressive Stiffness of the Mouse Lens: Strain and Morphometric Analyses. J Vis Exp. 2016; (111):53986. 10.3791/53986 27166880 PMC4942030

[97] Parreno J, Emin G, Vu MP, Clark JT, Aryal S, Patel SD, Cheng C. Methodologies to unlock the molecular expression and cellular structure of ocular lens epithelial cells. Front Cell Dev Biol. 2022; 10:983178. 10.3389/fcell.2022.983178 36176273 PMC9514789

[98] Cheng C, Gao J, Sun X, Mathias RT. Eph-ephrin Signaling Affects Eye Lens Fiber Cell Intracellular Voltage and Membrane Conductance. Front Physiol. 2021; 12:772276. 10.3389/fphys.2021.772276 34899394 PMC8656704

