# Supplementary material for: Canonical ligand-dependent and non-canonical ligand-independent EphA2 signaling in the eye lens of wild-type, knockout, and aging mice
Source: Aging (Albany NY). 2024 Oct 25;16(20):13039–75. doi: 10.18632/aging.206144 (PMC11552635; doi:10.18632/aging.206144)
Supplement: Supplementary Figures [file aging-16-206144-s001.pdf]

SUPPLEMENTARY FIGURES

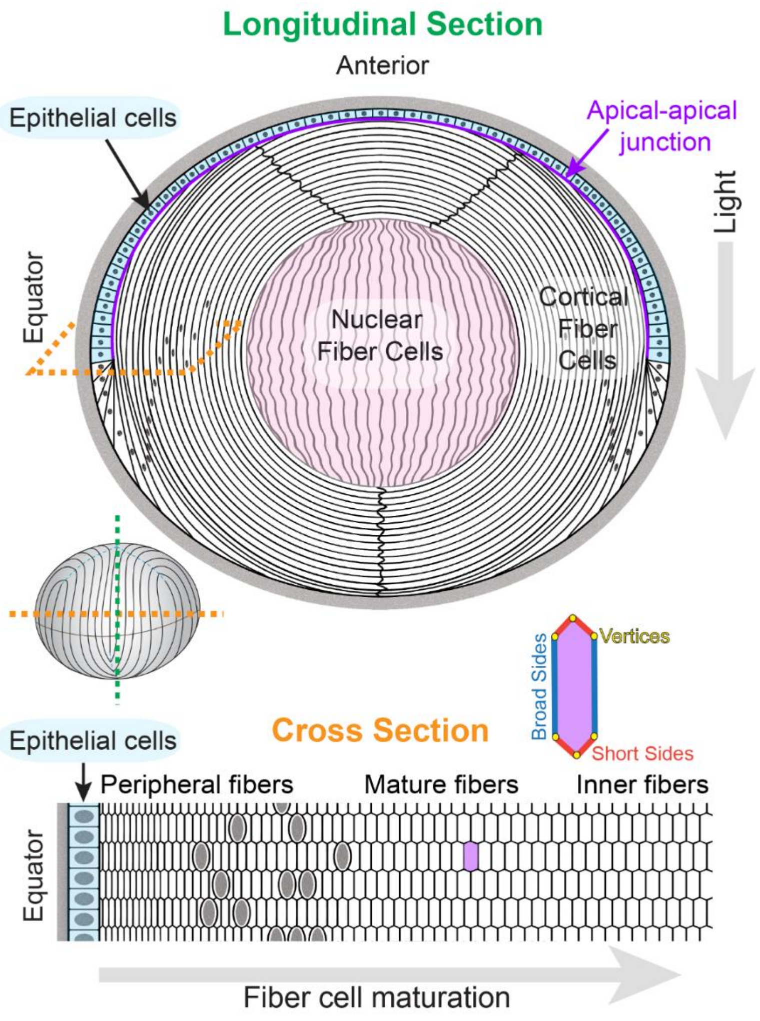

Supplementary Figure 1. Cartoon diagram depicting the lens when sectioned longitudinally (green) and in the cross orientation (orange). The lens is made up of two cell types: epithelial cells (blue) and fiber cells. The monolayer of epithelial cells covers the anterior hemisphere of the lens. Fiber cells can be further distinguished into cortical fiber cells (white) and nuclear fiber cells (pink). Due to the 3D organization of lens cells, there is an apical-apical junction (purple) between the epithelial cells and cortical fiber cells. Fiber cells are an elongated hexagon in cross section with 2 broad sides and 4 short sides. The cartoons are not drawn to scale. Modified from [1, 2].

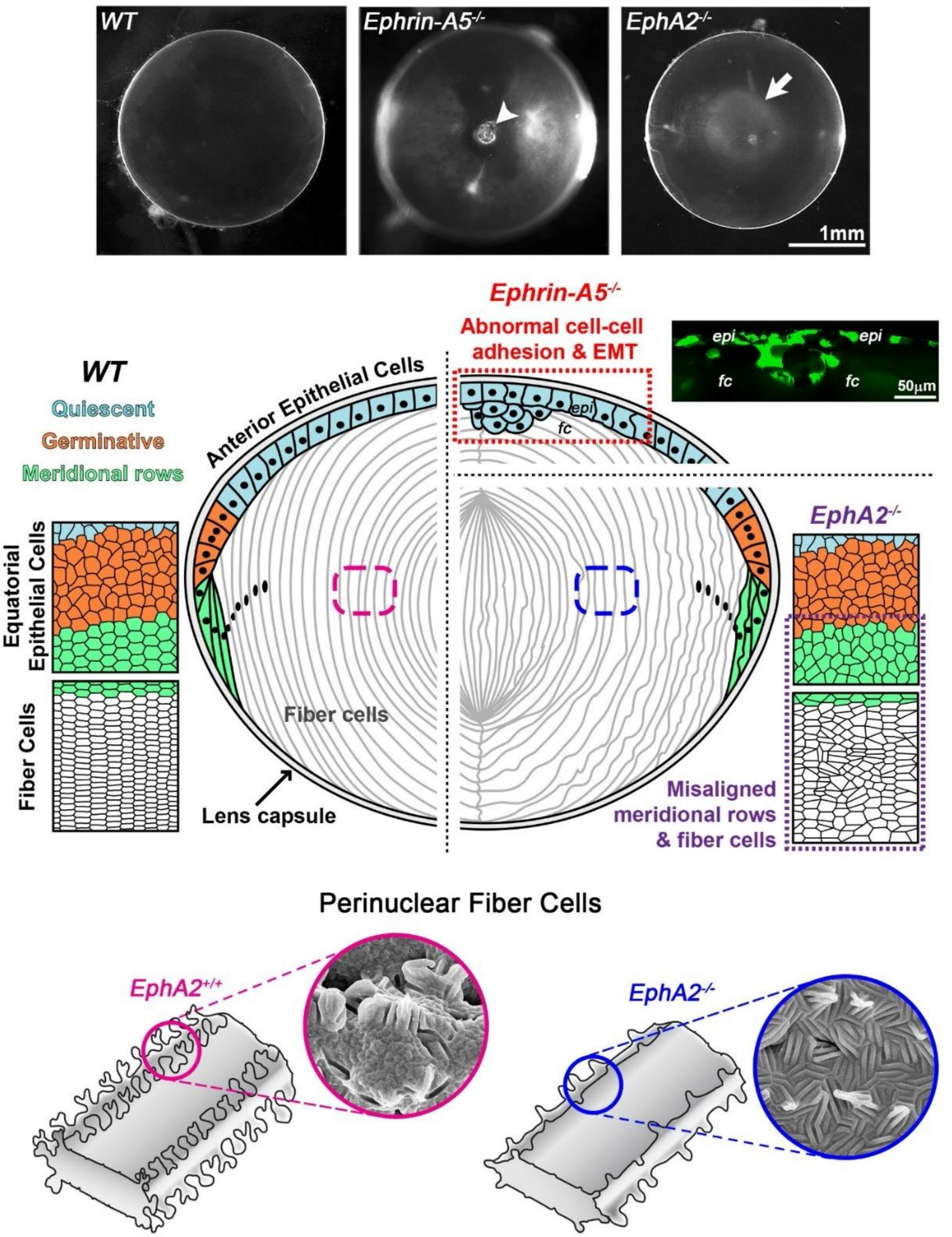

Supplementary Figure 2. A summary of *EphA2$^{-/-}$* and *ephrin-A5$^{-/-}$* lens phenotypes. Photos show a clear wild-type (*WT*) lens, an anterior cataract in the ephrin-A5$^{-/-}$ lens (arrowhead), and a nuclear cataract in *EphA2$^{-/-}$* lens (arrow). Scale bar, 1mm. In *WT* lenses, anterior epithelial cells (light blue) are quiescent, while equatorial epithelial cells (orange) will proliferate in the germinative zone. Equatorial epithelial cells organize into hexagon-shaped cells in neat meridional rows (green). Fiber cells retain this hexagonal and ordered cell packing. In *ephrin-A5$^{-/-}$* lenses, there are often anterior cataracts that are caused by epithelial-to-mesenchymal transition (EMT) and abnormal cell-cell adhesion in knockout anterior epithelial cells (red dashed box). The image to the right of the red dashed box is a 2D projection of a 3D scan through the anterior pole of a fresh green-fluorescent-protein-positive *ephrin-A5$^{-/-}$* lens. Scale bar, 50µm. In *EphA2$^{-/-}$* lenses, equatorial epithelial cells fail to form organized meridional rows of hexagon-shaped cells leading to disordered fiber cells (purple dashed box). Perinuclear fiber cells of WT lenses have globular membrane morphology and large interlocking protrusions with tongue-and-groove interdigitations (pink ellipse and circles). *EphA2$^{-/-}$* perinuclear fibers retain pronounced tongue-and-groove interdigitations with small protrusions that do not interlock with protrusions from neighboring cells (dark blue ellipse and circles). The cartoons are not drawn to scale. Modified from [3, 4].

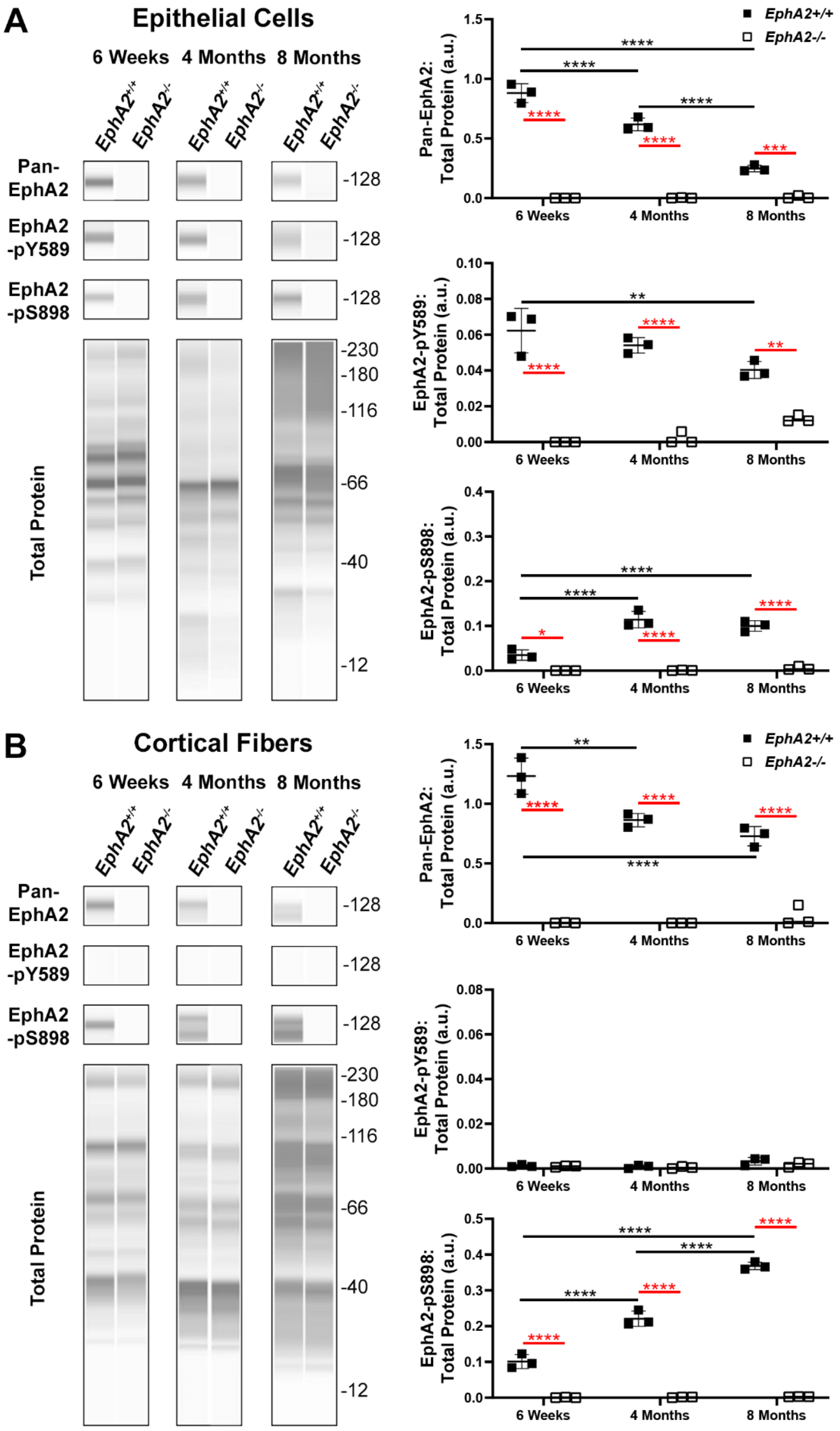

Supplementary Figure 3. JESS capillary-based Westerns demonstrate antibody specificity with lack of signal in *EphA2*$^{-/-}$ samples. Representative gel bands for pan-EphA2 (~128kDa), EphA2-pY589 (~128kDa), EphA2-pS898 (~128kDa), and total protein profiles (12-230kDa) in lens epithelial (A) and cortical fiber cell fractions (B) are shown. The dot plots display the average and standard deviation of amounts for proteins of interest normalized to the amount of total protein in each sample. Black horizontal lines mark changes between *EphA2*$^{+/+}$ samples at different ages. Red horizontal lines mark changes between *EphA2*$^{+/+}$ and *EphA2*$^{-/-}$ samples. *, P< 0.05; ** P < 0.01; *** P < 0.001; **** P < 0.0001. N = 3 biological replicates per group. (A) In epithelial cells, the amount of pan-EphA2 showed a decrease with age in *EphA2*$^{+/+}$ samples. There was a slight decrease in EphA2-pY589 protein levels between 6-week-old and 8-month-old *EphA2*$^{+/+}$ samples. The amount of EphA2-pS898 increased between 6-week-old and 4-month-old or 8-month-old *EphA2*$^{+/+}$ samples. (B) In cortical fiber samples, pan-EphA2 increased between 6-week-old and 4-month-old or 8-month-old *EphA2*$^{+/+}$ samples. No EphA2-pY589 proteins were detected in cortical fiber cells. The levels of EphA2-pS898 increased with age in *EphA2*$^{+/+}$ cortical fibers.

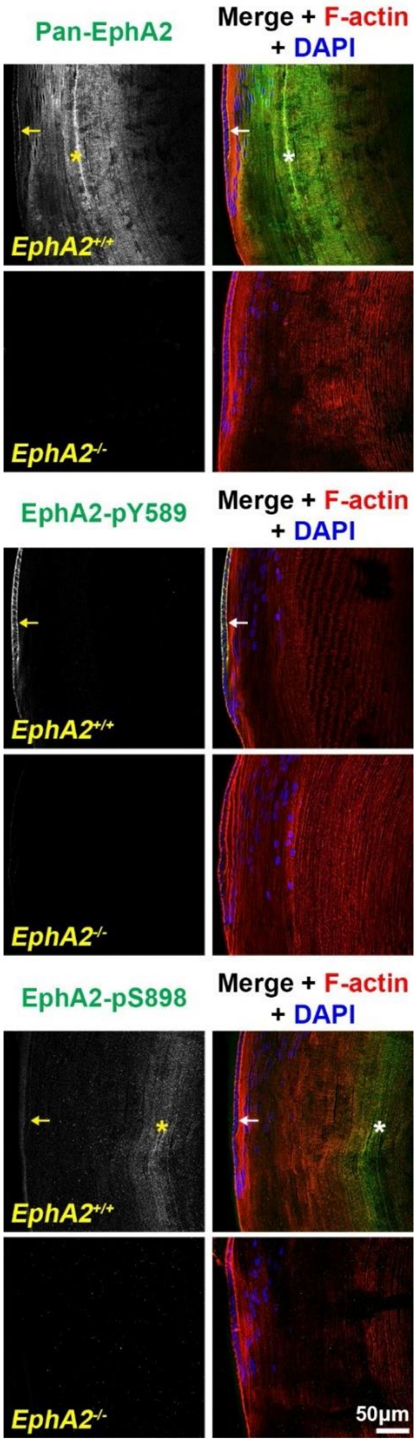

Supplementary Figure 4. Immunostaining of longitudinal sections from *EphA2$^{-/-}$* mice demonstrate antibody specificity with a lack of fluorescence signal. Longitudinal lens sections from 6-week-old *EphA2$^{+/+}$* and *EphA2$^{-/-}$* mice were stained with pan-EphA2 (green) antibody, EphA2-pY589 (green) antibody, or EphA-pS898 (green) antibody, and phalloidin (F-actin, red) and DAPI (nuclei, blue). No staining signals from the antibodies against EphA2 were observed in KO sections. Scale bar, 50µm.

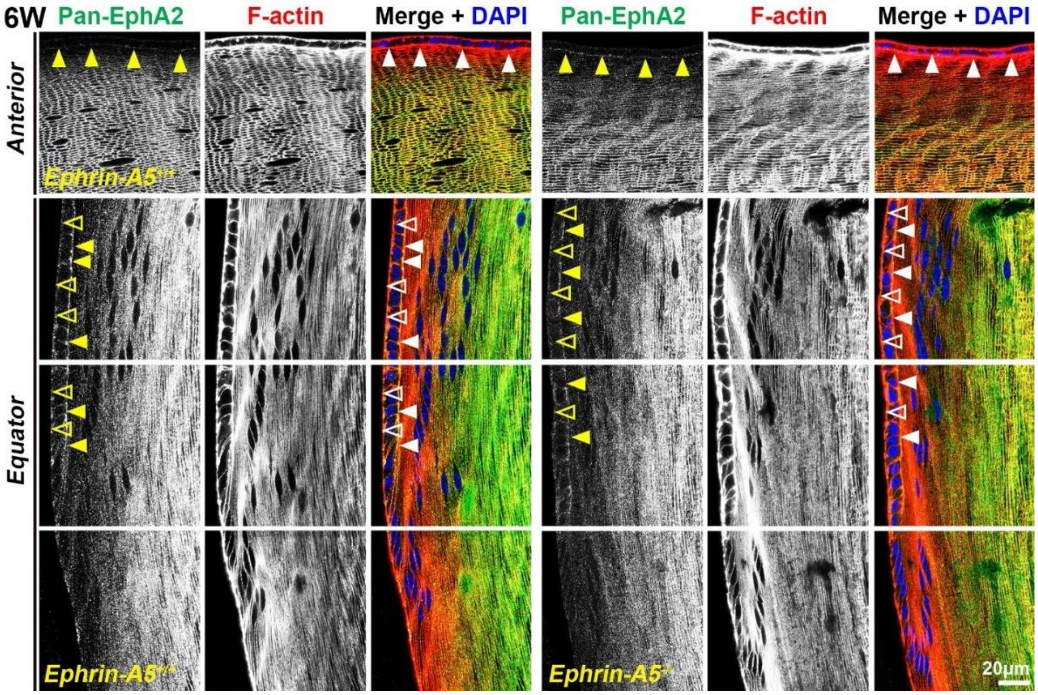

Supplementary Figure 5. In lens cells, pan-EphA2 is localized to the cell membrane. Longitudinal lens sections from 6-week-old control and *ephrin-A5*$^{-/-}$ mice were stained with pan-EphA2 (green) antibody, phalloidin (F-actin, red), and DAPI (nuclei, blue). Images of the equator region were taken in sequence along similar areas of the lens. Fluorescence signal from pan-EphA2 appeared along the cell membranes within the lens and was enriched at the apical-apical junction (arrowheads) between the epithelial and cortical fiber cells. Pan-EphA2 was present at the basal (open arrowheads) membranes and apical membranes (arrowheads) in equatorial epithelial cells. There was strong pan-EphA2 staining in cortical fiber cells. The staining patterns were comparable between control and *ephrin-A5*$^{-/-}$ samples. Scale bar, 20μm.

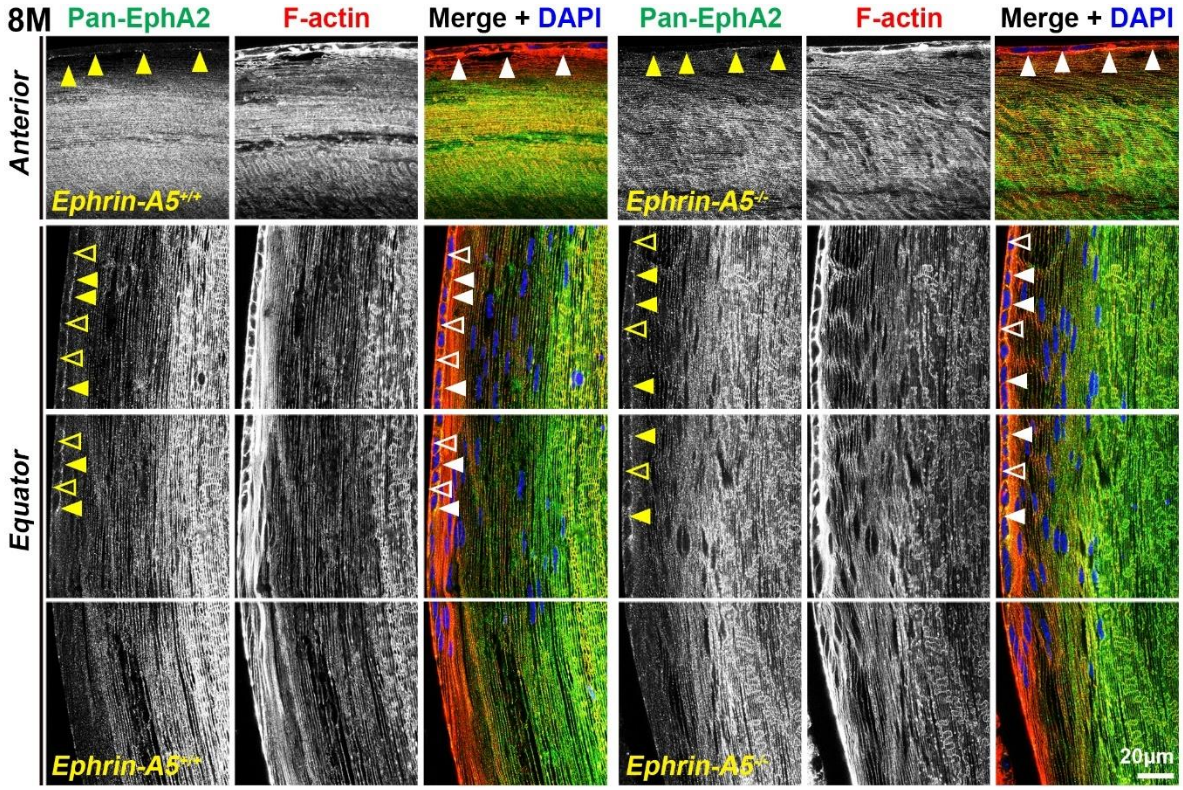

Supplementary Figure 6. In lens cells, pan-EphA2 is localized to the cell membrane. Longitudinal lens sections from 8-month-old control and *ephrin-A5*$^{-/-}$ mice were stained with pan-EphA2 (green) antibody, phalloidin (F-actin, red), and DAPI (nuclei, blue). Images of the equator region were taken in sequence along similar areas of the lens. Fluorescence signal from pan-EphA2 appeared along the cell membranes, and in anterior epithelial cells, there was enriched signal at the apical-apical junction (arrowheads). In equatorial epithelial cells, pan-EphA2 was enriched at the basal (open arrowheads) membranes and apical membranes (arrowheads). Pan-EphA2 was also found to outline the cortical fiber cells. These patterns were consistent between control and *ephrin-A5*$^{-/-}$ sections. Scale bar, 20μm.

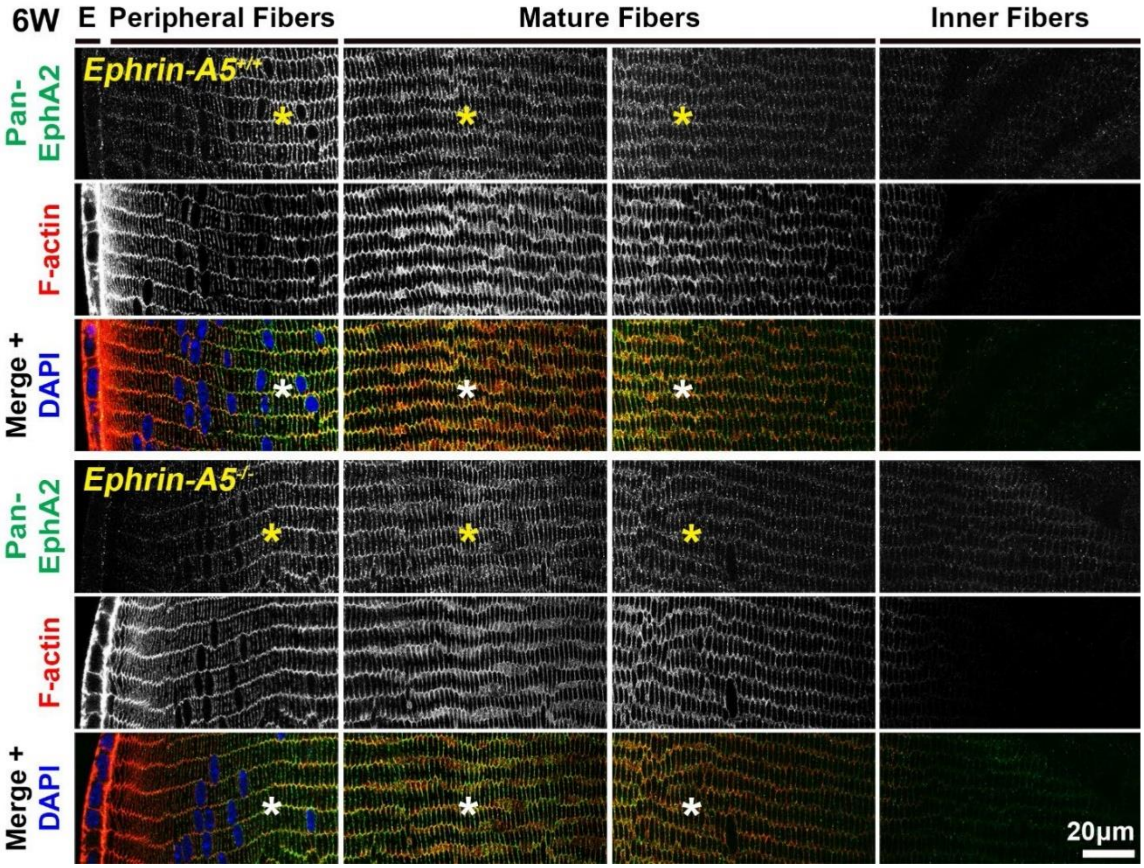

Supplementary Figure 7. In lens fiber cells, pan-EphA2 is localized to the cell membrane and enriched along the short sides. Lens sections in the cross orientation from 6-week-old control and *ephrin-A5*$^{-/-}$ mice were stained with pan-EphA2 (green) antibody, phalloidin (F-actin, red), and DAPI (nuclei, blue). E denotes the epithelial cells, and images of the fiber cells were taken in sequence along similar areas of the lens. Fluorescence from pan-EphA2 was found along the cell membranes of peripheral and mature fiber cells (asterisks) in the lens cortex region with slightly increased signal along the short sides of the cells. There was no difference in staining pattern between control and *ephrin-A5*$^{-/-}$ sections. Scale bar, 20µm.

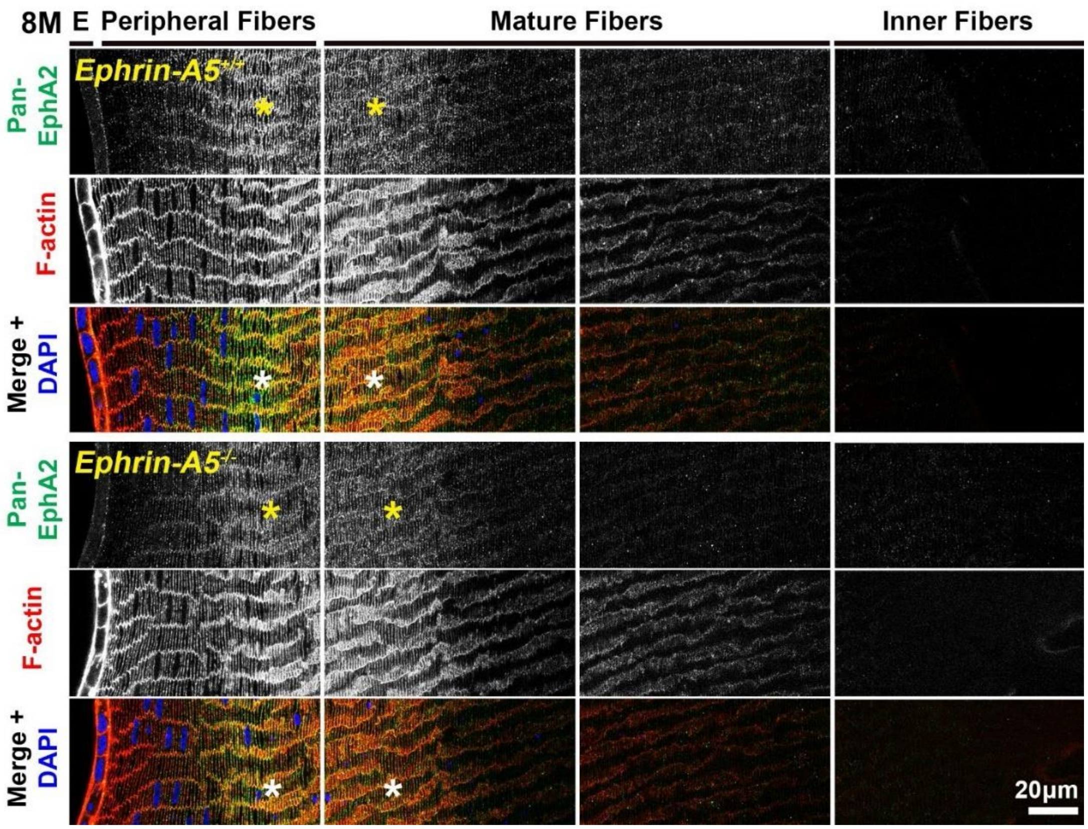

Supplementary Figure 8. In lens fiber cells, pan-EphA2 is localized to the cell membrane and enriched along the short sides. Lens sections in the cross orientation from 8-month-old control and *ephrin-A5*$^{-/-}$ mice were stained with pan-EphA2 (green) antibody, phalloidin (F-actin, red), and DAPI (nuclei, blue). E denotes the epithelial cells, and images of the fiber cells were taken in sequence along similar areas of the lens. Fluorescence from pan-EphA2 was found along the cell membranes of peripheral and mature fiber cells (asterisks) in the lens cortex region with slightly increased signal along the short sides of the cells. There was no difference in staining pattern between control and *ephrin-A5*$^{-/-}$ sections. Scale bar, 20µm.

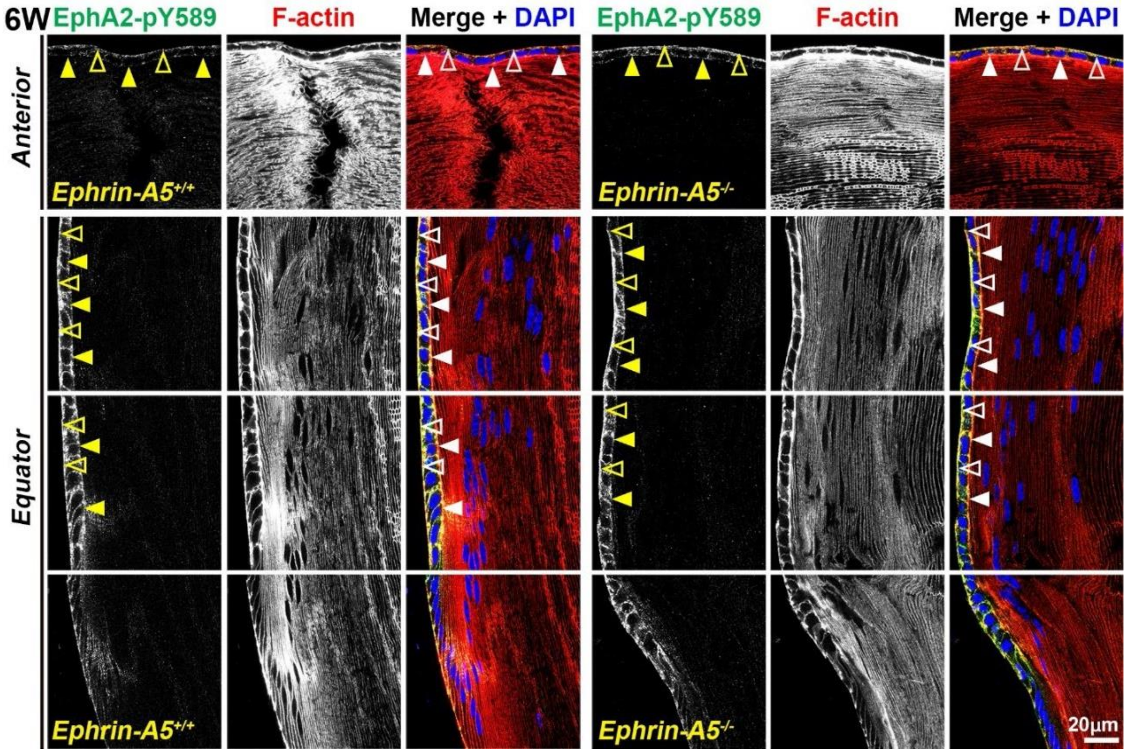

Supplementary Figure 9. Canonically active EphA2-pY589 protein is enriched at basal and apical membranes of lens epithelial cells. Longitudinal lens sections from 6-week-old control and *ephrin-A5*$^{-/-}$ mice were stained with pan-EphA2 (green) antibody, phalloidin (F-actin, red), and DAPI (nuclei, blue). Images of the equator region were taken in sequence along similar areas of the lens. EphA2-pY589 signal was weakly found in anterior epithelial cells with enrichment at the basal (open arrowheads) and apical (arrowheads) membranes. There were strong staining signals in equatorial epithelial cells. EphA2-pY589 was present between cells at the lateral membranes and enriched at the basal (open arrowheads) and apical (arrowheads) membranes. The staining signals were comparable between control and *ephrin-A5*$^{-/-}$ sections. Scale bar, 20μm.

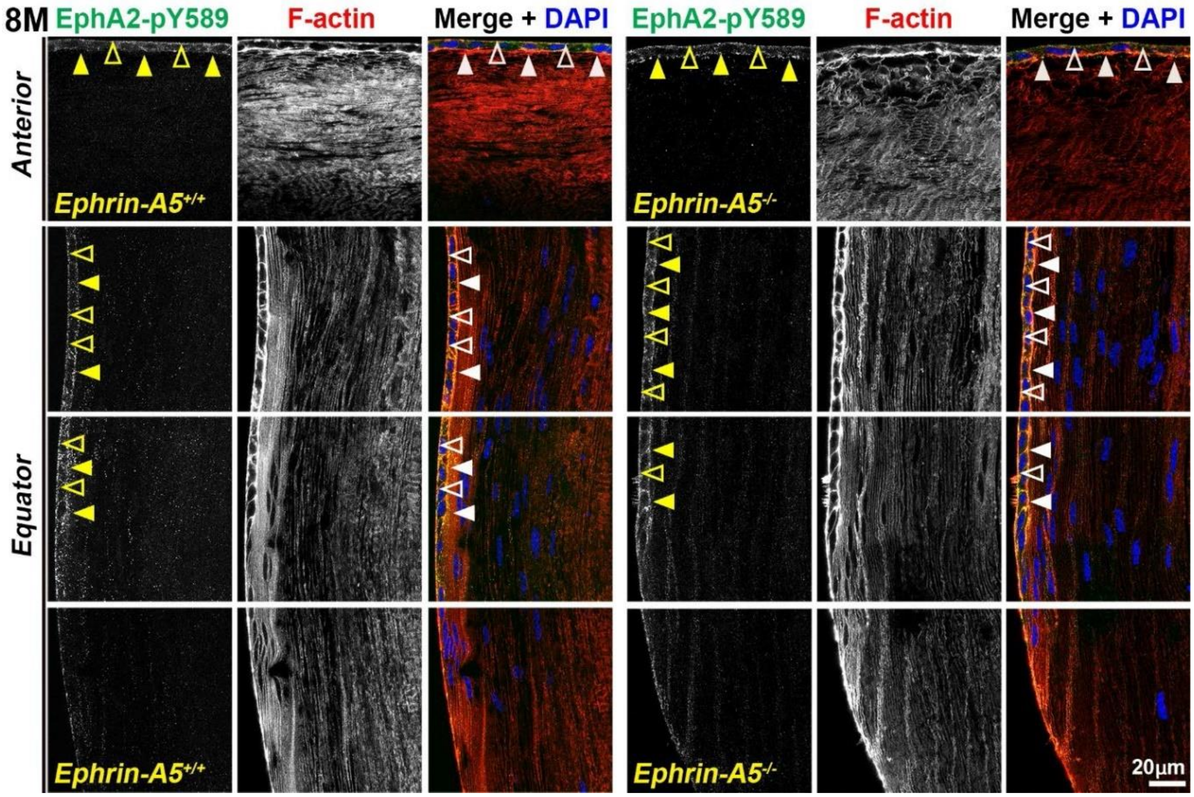

Supplementary Figure 10. Canonically active EphA2-pY589 protein is enriched at basal and apical membranes of lens epithelial cells. Longitudinal lens sections from 8-month-old control and *ephrin-A5*$^{-/-}$ mice were stained with pan-EphA2 (green) antibody, phalloidin (F-actin, red), and DAPI (nuclei, blue). Images of the equator region were taken in sequence along similar areas of the lens. EphA2-pY589 signal was weak in anterior epithelial cells and was stronger in equatorial epithelial cells. Staining signals were enriched at the basal (open arrowheads) and apical (arrowheads) membranes with staining also present at the lateral membranes between epithelial cells. Loss of ephrin-A5 did not obviously alter the EphA2-pY589 staining signal. Scale bar, 20µm.

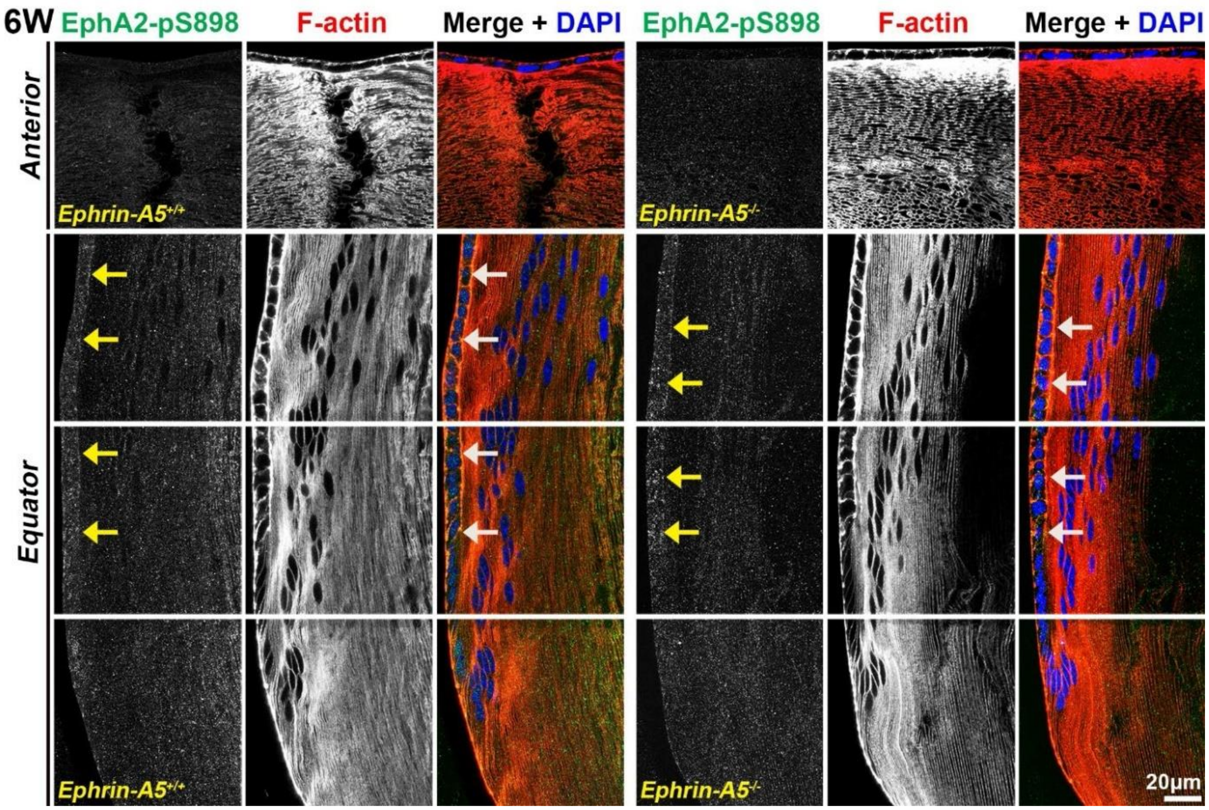

Supplementary Figure 11. Non-canonically active EphA2-pS898 protein is enriched in equatorial lens epithelial cells. Longitudinal lens sections from 6-week-old control and *ephrin-A5*$^{-/-}$ mice were stained with EphA2-pS898 (green) antibody, phalloidin (F-actin, red), and DAPI (nuclei, blue). Images of the equator region were taken in sequence along similar areas of the lens. Mature fibers cells with strong EphA2-pS898 cannot be seen in these images of the peripheral fibers. EphA2-pS898 signal was weak in anterior epithelial cells, but staining was stronger in equatorial epithelial cells (arrows). The staining signals were comparable between control and *ephrin-A5*$^{-/-}$ sections. Scale bar, 20µm.

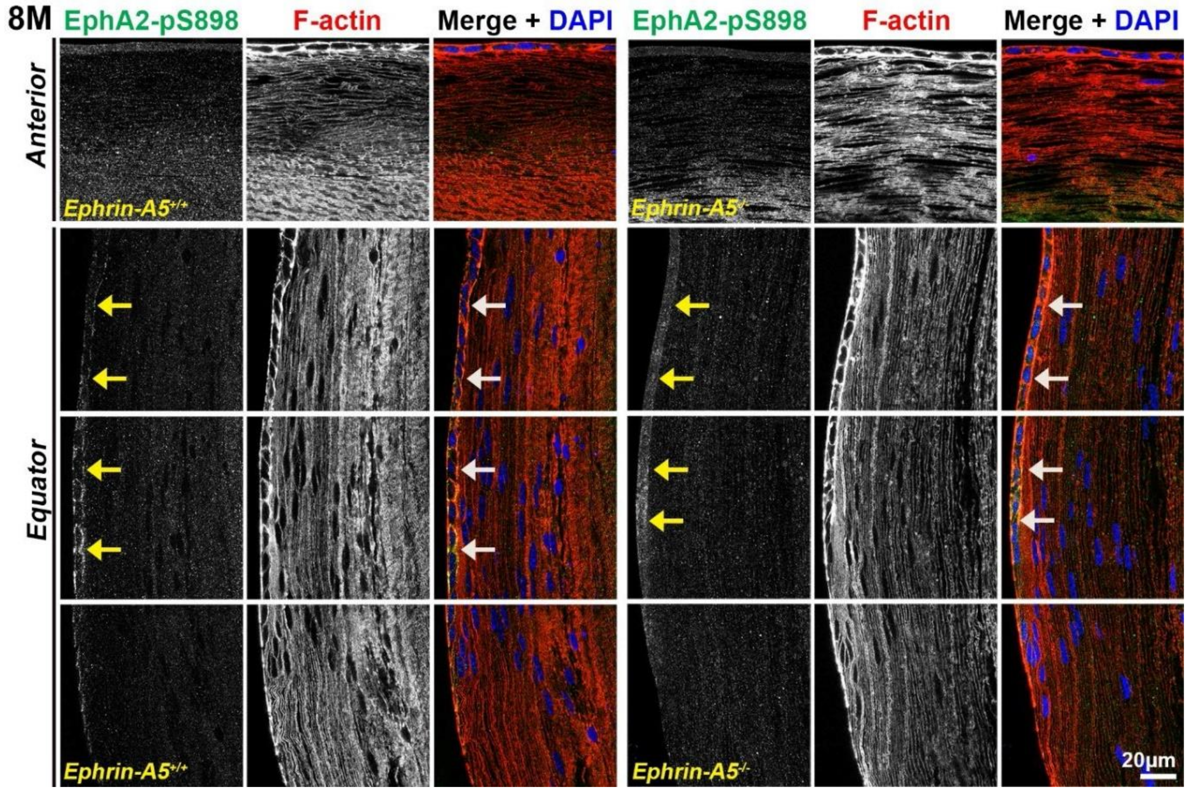

Supplementary Figure 12. Non-canonically active EphA2-pS898 protein is enriched in equatorial lens epithelial cells. Longitudinal lens sections from 8-month-old control and *ephrin-A5*$^{-/-}$ mice were stained with EphA2-pS898 (green) antibody, phalloidin (F-actin, red), and DAPI (nuclei, blue). Images of the equator region were taken in sequence along similar areas of the lens. Mature fiber cells with strong EphA2-pS898 cannot be seen in these images of the peripheral fibers. EphA2-pS898 signal was weak in anterior epithelial cells when compared to equatorial epithelial cells (arrows). The staining signals were comparable between control and *ephrin-A5*$^{-/-}$ sections. Scale bar, 20µm.

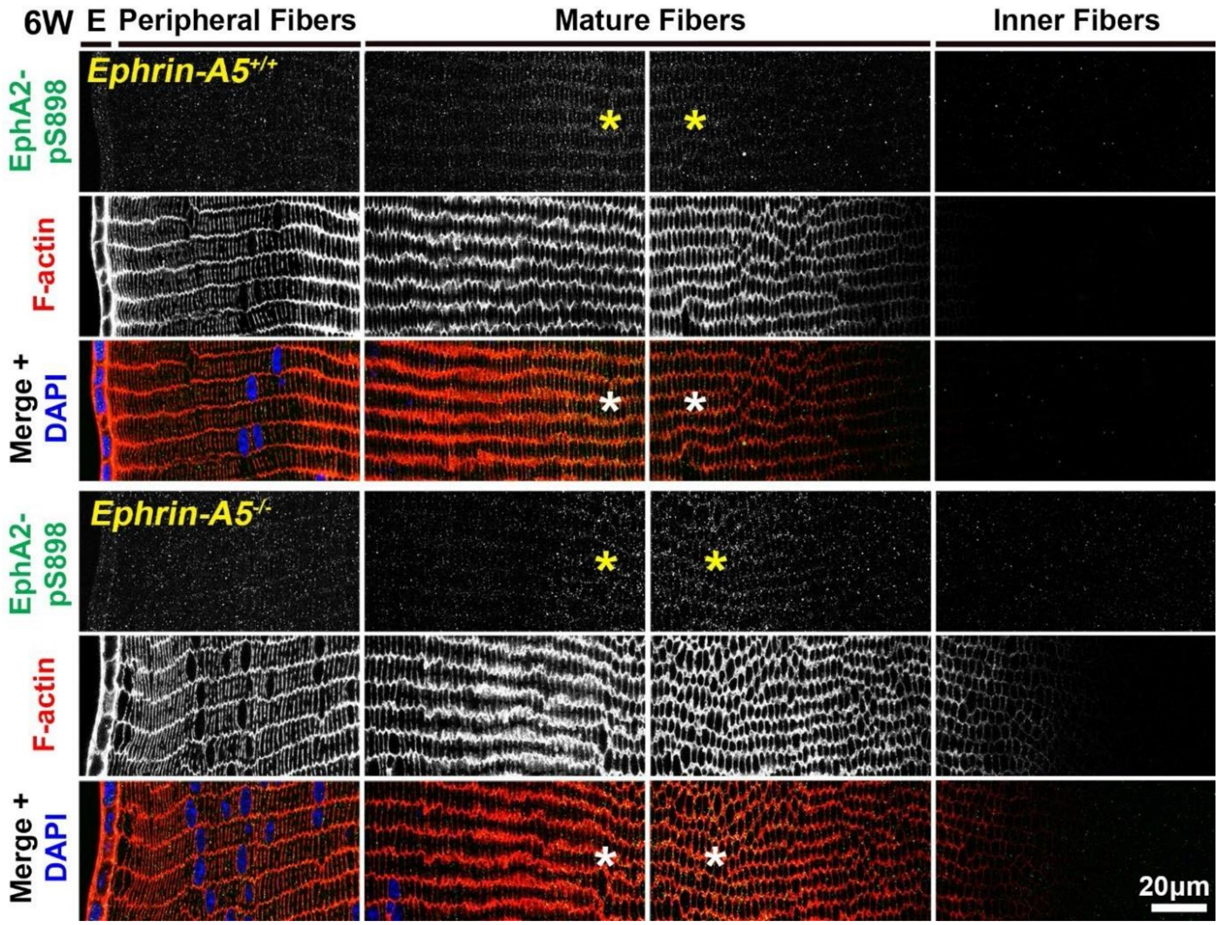

Supplementary Figure 13. In lens fiber cells, EphA2-pS898 signal is localized to the cell membrane of mature fibers. Lens sections in the cross orientation from 6-week-old control and *ephrin-A5*$^{-/-}$ mice were stained with EphA2-pS898 (green) antibody, phalloidin (F-actin, red), and DAPI (nuclei, blue). E denotes the epithelial cells, and images of the fiber cells were taken in sequence along similar areas of the lens. EphA2-pS898 signal was present at the membrane of mature fiber cells (asterisks). The signal appeared to be evenly distributed around the membrane without preference for the broad or short sides. The staining signals were comparable between control and *ephrin-A5*$^{-/-}$ sections. Scale bar, 20μm.

REFERENCES

1. Cheng C, Nowak RB, Amadeo MB, Biswas SK, Lo WK, Fowler VM. Tropomyosin 3.5 protects the F-actin networks required for tissue biomechanical properties. J Cell Sci. 2018; 131:jcs222042. https://doi.org/10.1242/jcs.222042 PMID:30333143

2. Cheng C, Parreno J, Nowak RB, Biswas SK, Wang K, Hoshino M, Uesugi K, Yagi N, Moncaster JA, Lo WK, Pierscionek B, Fowler VM. Age-related changes in eye lens biomechanics, morphology, refractive index and transparency. Aging (Albany NY). 2019; 11:12497–531. https://doi.org/10.18632/aging.102584 PMID:31844034

3. Cheng C, Fowler VM, Gong X. EphA2 and ephrin-A5 are not a receptor-ligand pair in the ocular lens. Exp Eye Res. 2017; 162:9–17. https://doi.org/10.1016/j.exer.2017.06.016 PMID:28648759

4. Cheng C, Wang K, Hoshino M, Uesugi K, Yagi N, Pierscionek B. EphA2 Affects Development of the Eye Lens Nucleus and the Gradient of Refractive Index. Invest Ophthalmol Vis Sci. 2022; 63:2. https://doi.org/10.1167/iovs.63.1.2 PMID:34978559
